# Evaluating cytokine-based therapeutics in human 3D *in vitro* immune-containing assembloids

**DOI:** 10.3389/fcell.2026.1930651

**Published:** 2026-10-15

**Authors:** Nina Maenhoudt, Mechthild Lütge, Melanie Obenloch, Aljona Caironi, Maria Kiratzis, Pol Jean-Mairet, Samuele Graziano, Philipp Fröbel, Tamara Hüsser, Emilio Yángüez, Maria Antsiferova, Adrian M. Filip, Christian Müller, Stephane Leclair, Stephan Gasser, Pablo Umaña, Sara Colombetti, Floriana Cremasco, Samuele Calabro

**Affiliations:** 2 Roche Innovation Center Zurich, Roche Pharma Research and Early Development, F. Hoffmann-La Roche, Schlieren, Switzerland; 2 Roche Innovation Center Basel, Roche Pharma Research and Early Development, F. Hoffmann-La Roche, Basel, Switzerland; 3 Computational Sciences Center of Excellence, F. Hoffmann-La Roche, Basel, Switzerland; 4 Institute of Human Biology (IHB), Roche Pharma Research and Early Development, F. Hoffmann-La Roche, Basel, Switzerland; 5 Roche Innovation Center Munich, Roche Pharma Research and Early Development, F. Hoffmann-La Roche, Penzberg, Germany

**Keywords:** assembloids, cancer immunotherapy, cancer-associated fibroblast (CAF), colorectal cancer, interferon-gamma (IFNg), next-generation cytokines, organoids, tumor microenvironment (TME)

## Abstract

**Introduction:**

Cancer immunotherapies require accurate preclinical models that recapitulate the complex cellular interactions within the human tumor microenvironment. While patient-derived organoids have emerged as powerful tools for cancer research, standard cultures lack the stromal and immune compartments necessary to evaluate immunotherapeutic mechanisms. This limitation hampers the development of next-generation targeted cytokine therapies.

**Methods:**

We applied a 3D immune-containing colorectal cancer assembloid platform integrating patient-derived organoids, cancer-associated fibroblasts and allogeneic peripheral blood mononuclear cells within a collagen matrix. We characterized cellular responses to interferon-gamma (IFNg) and engineered IFNg receptor agonists targeted to fibroblast activation protein (FAP) through single-cell transcriptomics, immunohistochemistry, cytokine profiling, and functional T-cell migration assays.

**Results:**

The assembloid model successfully recapitulated key features of IFNg signaling, including tissue growth suppression, enhanced chemokine secretion (CXCL10), and CD8^+^ T-cell recruitment. Single-cell transcriptomics revealed that fibroblasts were the most responsive cell population, undergoing a phenotypic transition from myofibroblasts toward antigen-presenting CAFs (apCAFs), characterized by robust upregulation of MHC class II genes. FAP-targeted IFNg molecules exhibited attenuated activity compared to recombinant IFNg, successfully avoiding upregulation of the severe immunosuppressive checkpoint molecule PD-L1.

**Discussion:**

This 3D assembloid model provides a physiologically relevant platform for dissecting complex tumor-immune interactions and optimizing targeted immunotherapies. This approach enables the study of epithelial-stromal-immune crosstalk in cancer and offers a robust preclinical framework for screening and mechanistically validating next-generation targeted cytokine therapies.

## 1 Introduction

Cancer immunotherapy has revolutionized the clinical landscape in oncology by harnessing the immune system to target malignancies, yet clinical success remains highly variable across patient populations (Chuprin et al., 2023). Colorectal cancer (CRC), the third most common cancer worldwide, exemplifies this challenge (Boukouris et al., 2022). While immune checkpoint inhibitors demonstrate remarkable efficacy in microsatellite instability-high (MSI-H) tumors, the majority of CRC patients with microsatellite stable (MSS) disease show limited response to current immunotherapeutic strategies (Boukouris et al., 2022). This therapeutic gap underscores the urgent need for next-generation cancer immunotherapies, including engineered cytokines, bispecific antibodies, and adoptive cell therapies, which promise enhanced tumor selectivity and immune activation (Garg et al., 2024).

The development of these therapeutics faces a significant translational challenge: while established preclinical models provide essential insights, there remains a critical need for human-based platforms that more faithfully recapitulate the complex and dynamic interactions within the tumor microenvironment (TME) (Wang et al., 2022). The TME comprises a heterogeneous ecosystem of malignant cells, stromal components, immune and endothelial cells, and extracellular matrix, all of which collectively influence therapeutic response and resistance (Devarasetty et al., 2020). Traditional two-dimensional (2D) cell culture systems fail to capture this spatial and cellular complexity, while animal models, despite their proven relevance, often exhibit species-specific immune responses that poorly predict human clinical outcomes (Kapałczyńska et al., 2018). Patient-derived xenografts (PDXs) require immunocompromised hosts, precluding the study of immunotherapy mechanisms, whereas syngeneic mouse models rely on murine tumor cell lines that diverge significantly from human tumor biology (Bareham et al., 2021; Wang et al., 2022). Even though humanized mouse models advance the study of therapies like T-cell bispecific antibodies, the host’s murine TME remains a major bottleneck that complicates human translation (Bareham et al., 2021).

Over the past decade, three-dimensional (3D) patient derived organoid models have emerged as powerful research tools to overcome these hurdles (Drost and Clevers, 2018). Organoids are self-forming 3D tissue reconstructions that reproduce key histological, functional, and genetic aspects of the tissue of origin. They show high expansion capacity and have been successfully used to establish living biobanks for various malignancies, including colorectal cancer (Drost and Clevers, 2018). However, standard organoid cultures lack the stromal and immune compartments essential for evaluating immunotherapeutic interventions (Wang et al., 2022). To reach the full potential of these *in vitro* models, recent efforts by Filip et al. and others have focused on establishing complex composite models, often termed “assembloids” (Filip et al., 2026; Li et al., 2026). By combining epithelial organoids with stromal components (such as fibroblasts) and immune cells, assembloids better mimic the architectural organization and cellular crosstalk present in the native tissue environment (Filip et al., 2026; Li et al., 2026). Yet, validating these established platforms for complex immunotherapeutic evaluation and leveraging them to uncover stromal dynamics remain largely unexplored.

In this study, we apply and validate the patient-derived CRC assembloid platform to map the cell-type-specific mechanics of interferon-gamma (IFNg) and evaluate targeted cytokine therapies. Specifically, we leverage this system to characterize the mechanisms of action (MoA) of IFNg, a master regulator of cell-mediated immunity and known for its potential to orchestrate anti-tumor responses and altering the tumor stroma (Jorgovanovic et al., 2020; Liu, 2025). However, IFNg exhibits a complex dual biological role: while driving anti-tumor mechanisms such as T-cell recruitment and antigen presentation, it paradoxically triggers adaptive immune resistance through the rapid induction of immunosuppressive checkpoints, most notably PD-L1 (Ivashkiv, 2018; Gocher et al., 2022). To circumvent these detrimental feedback loops alongside the limitations associated with the systemic administration of IFNg, such as severe dose-limiting toxicities, we engineered an attenuated tumor-targeted variant by conjugating IFNg to an antibody fragment specific for Fibroblast Activation Protein (FAP). Our studies demonstrate that assembloid models were able to effectively capture these pleiotropic effects of IFNg, including the induction of PD-L1 alongside the phenotypic remodeling of CAFs. We subsequently use these novel models to evaluate the functional activity of FAP targeted-IFNg molecules, providing a biologically relevant platform to assess targeted immune recruitment and modulation within a heterogeneous human TME. Collectively, these findings demonstrate the utility and have strengthened the use of the assembloid model as a translational tool to dissect nuanced stromal-immune interactions and accelerate the optimization of targeted cancer immunotherapies.

## 2 Results

### 2.1 Characterization of a CRC immune-containing assembloid model

The 3D immune-containing model, developed and described by Filip et al. (2026), combines patient-derived CRC organoids, autologous cancer-associated fibroblasts (CAFs), and allogeneic peripheral blood mononuclear cells (PBMCs) within a collagen matrix, using a hydrogel-in-oil emulsion system (Figure 1A). This approach generates a compact structure, referred to as assembloid, where all the different cell types are spatially organized to mimic the cellular heterogeneity and architecture of the tumor microenvironment (Figure 1B; Supplementary Figure S1A). Expanding on the findings of Filip et al., who showed a growing hypoxic and necrotic core inside assembloids, we performed a scaling analysis to determine the optimal balance between tissue complexity and metabolic stability. By proportionally scaling both the construct volume and the absolute cell number, doubling the input for 10 uL and quadrupling it for 20 uL droplets, we observed significant size-dependent effects on tissue health. While these larger assembloids successfully increased total tissue mass, the expanded physical dimensions resulted in prominent necrotic cores and morphological shifts in the CAF compartment (Supplementary Figures S1B,C). These results suggest that the increased volume and cellular density of the larger constructs likely result in diffusion-limited oxygen and nutrient gradients (Li et al., 2026). While the baseline 5 uL architecture is sufficient to capture translatable features of tumor hypoxia, and because preserving optimal cellular health and morphology is critical for evaluating specific stromal-immune responses, we utilized the 5 uL scale to ensure a viable, highly responsive TME landscape for all downstream screening.

**FIGURE 1:**
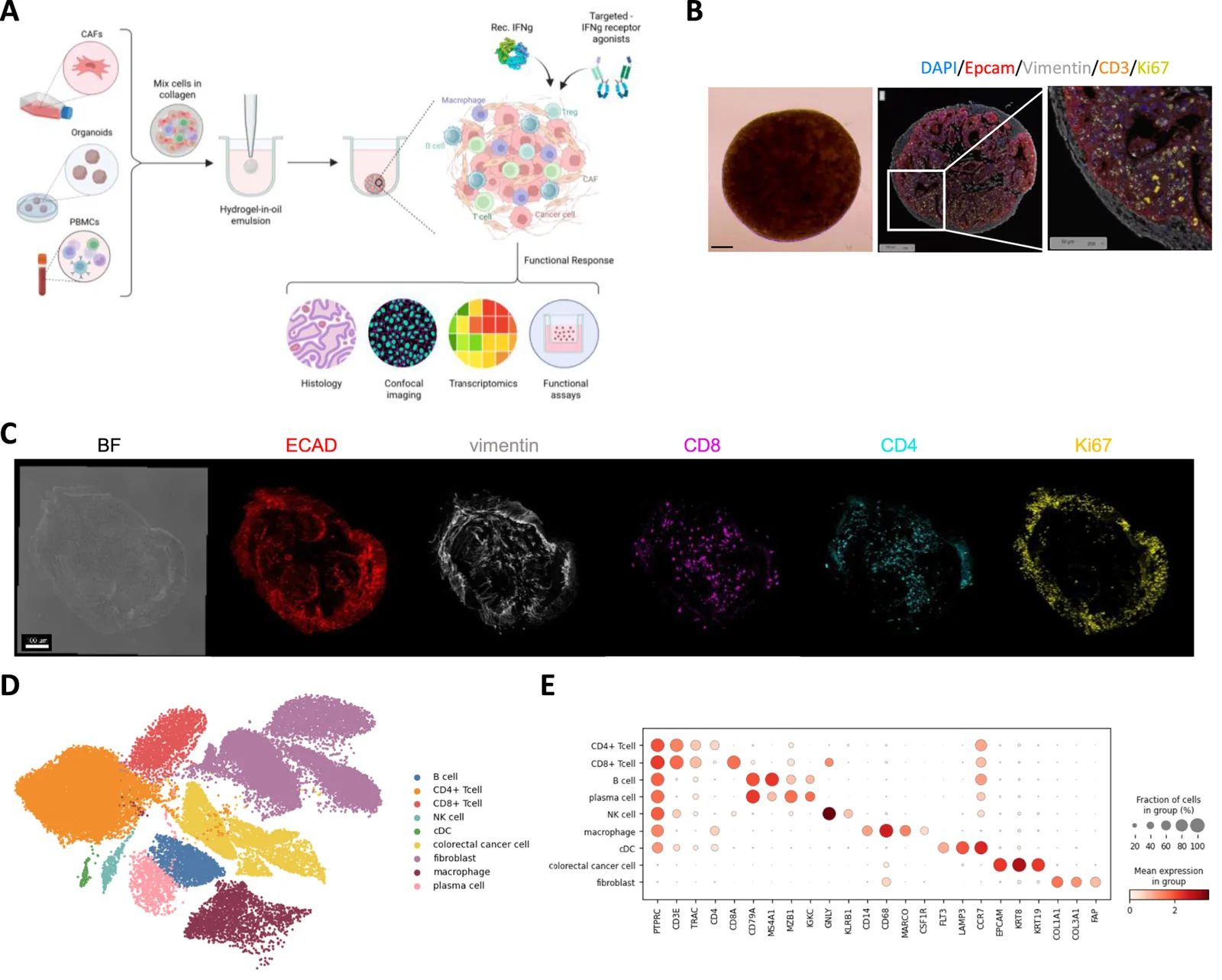
Colorectal assembloid generation and characterization. **(A)** Schematic of CRC assembloid generation. **(B)** Brightfield image of assembloids at day 6. Scale bar, 100 μm. Representative immunofluorescence staining of the assembloid showing DAPI (nuclei, blue), EpCAM (epithelial cells, red), Vimentin (fibroblasts, white), CD3 (T cells, yellow), and Ki67 (proliferation marker, yellow). Scale bars indicate 100 μm (left) and 50 μm (right). **(C)** Representative images of cellular composition and organization within the assembloid, showing BF (bright field), ECAD (red), Vimentin (grey), CD8 (purple), CD4 (cyan), and Ki67 (yellow). Scale bar: 100 μm. **(D)** UMAP visualization of single cell gene expression with cells colored by cell identity, aggregated from assembloids across n = 6 independent patients (biological replicates). **(E)** Dot plot showing marker gene expression for each cell cluster, confirming the identity of the distinct populations present in the assembloid. Panel **(A)** created with BioRender.com.

Whole-mount multiplex imaging and immunohistological analysis of the assembloids confirmed the successful co-engraftment and spatial organization of the diverse cell types. Imaging revealed large E-cadherin+ (ECAD) epithelial cancer organoid structures embedded within a dense network of Vimentin + CAFs. Importantly, spatial distribution analysis demonstrated that while CD8^+^, and CD4^+^ T lymphocytes and CD68^+^ macrophages successfully infiltrated the tumor-dense areas, the majority of the immune cells preferentially localized within the stroma-rich areas of the construct (Figures 1C,D; Supplementary Figures S1D,E). These findings confirmed the incorporation of relevant cell populations and their spatial arrangement, reflecting the structural complexity of the TME. To further decipher the cellular complexity and molecular phenotype of the model, we performed single-cell RNA-sequencing (scRNA-seq) on assembloids. Unsupervised clustering identified distinct cell clusters corresponding to the input populations (Figure 1D). We successfully detected colorectal cancer cells and fibroblasts alongside a highly diverse immune compartment encompassing CD4^+^ T cells, CD8^+^ T cells, B cells, plasma cells, natural killer (NK) cells, macrophages, and dendritic cells (DCs) (Figure 1D). Assessment of the relative abundance of these cell types across multiple untreated patient-derived lines demonstrated the model’s ability to capture inherent inter-patient heterogeneity within the immune and stromal compartments (Supplementary Figure S2A). Profiling of the immune compartment revealed a diverse composition of immune cell types across all six patient-derived lines, encompassing CD4^+^ T cells, CD8^+^ T cells, B cells, plasma cells, NK cells, macrophages, and conventional dendritic cells (cDCs) (Supplementary Figure S2B). Gene expression analysis validated the cluster identities (Figure 1E; Supplementary Figure S2C). Colorectal cancer cells robustly expressed epithelial markers (EPCAM, KRT8, KRT19), fibroblasts were defined by mesenchymal and CAF-associated genes (COL1A1, COL3A1, FAP), and the diverse immune clusters faithfully retained their specific molecular signatures (Figure 1E; Supplementary Figure S2C).

### 2.2 Assembloids recapitulate key features of the IFNg response

Having established the baseline multicellular complexity of the patient-derived assembloids and their potential to recapitulate the human tumor microenvironment, we next evaluated their capacity to show structural, molecular and functional changes to IFNg. Histological assessment via hematoxylin and eosin (H&E) staining revealed that IFNg treatment resulted in a significant growth suppression, evidenced by a marked reduction in the overall tissue area of the assembloids (Figure 2A). A significant reduction in proliferation in IFNg-treated models compared to untreated controls could also be observed, as quantified by the reduced percentage of Ki67-positive tissue area (Figure 2B). IFNg is known to induce chemokines release including the T-cell chemoattractant CXCL10. We therefore analyzed the conditioned media of the assembloids treated with IFNg, for secretion of CXCL10. As expected, a dose-dependent increase in CXCL10 levels was observed following IFNg treatment (Figure 2C). In addition, we observed patient-specific differences in CXCL10 secretion, highlighting variability in how different patient-derived assembloids respond to IFNg (Figure 2C). To further investigate the functional relevance of CXCL10 secretion, a transwell migration assay was designed (Figure 2D). Two assembloids, generated from the same patient material, were placed in the lower chamber with or without treatment, and CMFDA-labeled naive CD8^+^ T cells were added to the upper compartment. T cells were allowed to migrate to the lower chamber over 48 h, after which the supernatant containing migrated CD8^+^ T cells was collected and quantified using flow cytometry. Analysis confirmed that conditioned media from IFNg-treated assembloids significantly enhanced CD8^+^ T-cell migration compared to untreated controls (Figure 2E). While this enhanced CD8^+^ T cell migration was a conserved hallmark of the IFNg response, we observed inherent biological variability in the magnitude of immune cell recruitment across different independent patient lines (Supplementary Figure S3A). Broader multiplex cytokine profiling of the conditioned media of assembloids treated with recombinant IFNg confirmed, along with CXCL10 release, the transition to a highly inflamed microenvironment, characterized by significant elevations in the secretion of pro-inflammatory cytokines including IL-1α, TNFα, IL-12 and CCL2 (Figure 2F). Furthermore, IL-6, a well-known stromal-immune crosstalk driver, was consistently, albeit non-significantly, elevated upon IFNg treatment (Supplementary Figure S3B). An extended panel of cytokines and growth factors (such as CCL3, CCL4, CCL5, and CCL7) highlighted distinct, patient-specific patterns of cytokine induction, underscoring the platform’s utility in capturing inter-patient heterogeneity (Supplementary Figure S3C). A critical downstream consequence of IFNg signaling within the TME is the induction of antigen presentation and localized immune evasion feedback loops. Immunohistochemical analysis confirmed that IFNg treatment potently drove these phenotypes, resulting in a significant, widespread upregulation of both MHC-I and the immune checkpoint molecule PD-L1 in the assembloids, although predominantly in stromal regions (Figure 2G; Supplementary Figure S3D). Assessment across multiple independent patient lines validated these findings, demonstrating consistent upregulation of both MHC-I and PD-L1 while the model captured donor-specific differences in response levels (Supplementary Figure S3E). Collectively, these data demonstrate that the complex 3D assembloid models successfully recapitulate key features of IFNg signaling, characterized by altered tissue proliferation, immune cell recruitment, and the induction of immunosuppressive checkpoints, while faithfully preserving the intrinsic heterogeneity of individual patient responses. This underscores its potential as a valuable platform for investigating IFNg-mediated mechanisms in a physiologically relevant context. Ultimately, this platform serves as a versatile preclinical model for screening novel immunotherapeutics, facilitating the early-stage identification of effective drug candidates and their specific modes of action.

**FIGURE 2:**
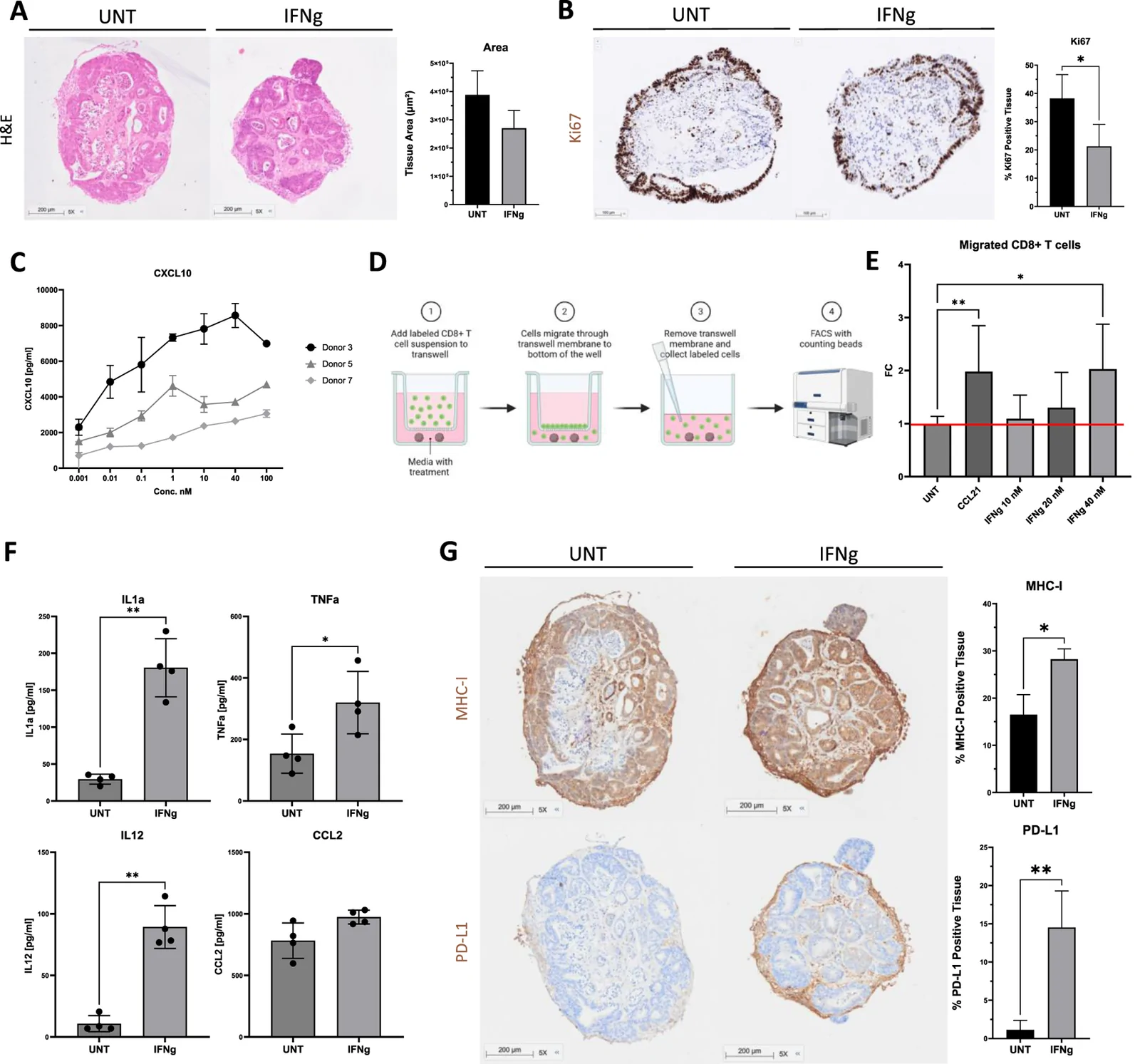
Evaluation of the 3D immunocompetent model to predict IFNg response. **(A)** Hematoxylin and eosin staining of untreated (UNT) and IFNg-treated assembloid shows a reduction in total tissue area following IFNg treatment (40 nM). Quantification of total area is shown as a bar graph. Data are presented as mean ± SD (n = 3 independent patients (biological replicates)). Scale bars, 200 μm. **(B)** Representative immunohistochemistry staining for Ki67 reveals a reduction in proliferative activity in IFNg-treated (40 nM) assembloids compared to untreated controls. Quantification of Ki67-positive cells is shown as a bar graph with mean ± SD (n = 4 independent patients (biological replicates), paired t-test). Scale bars, 100 μm. **(C)** Quantification of CXCL10 secretion in response to increasing doses of IFNg, as measured by ELISA in conditioned media. Each line represents an independent patient-derived assembloid line (Donor 3, 5 and 7). Data are shown as mean ± SD. **(D)** Schematic representation of a transwell migration assay to assess CD8+ T-cell recruitment. **(E)** Quantification of migrated CD8+ T cells in response to media with different treatments. IFNg-treated assembloids significantly enhanced CD8+ T-cell migration compared to untreated controls in a dose-dependent manner. CCL21 (0.1 ug/mL) was used as a positive control. Data are pooled from three independent patient-derived assembloid lines, normalized to baseline migration (red line) and displayed as mean ± SD, Mixed-effects model fitted by Restricted Maximum Likelihood (REML) with Dunnett’s post-hoc test. **(F)** Multiplex cytokine analysis reveals increased levels of inflammatory and immune-modulating cytokines, including IL-1a, TNFα, CCL2, and IL-12, in conditioned media from IFNg- treated (40 nM) assembloids. Data are presented as mean ± SD (n = 4 independent patients (biological replicates), paired t-test). **(G)** Immunohistochemical staining for MHC-I and PD-L1 demonstrates upregulation of these immune markers in IFNg-treated (40 nM) assembloids. Corresponding quantification of MHC-I and PD-L1 expression is represented as the percentage of positive-stained area (mean ± SD, n = 3 independent patients (biological replicates), paired t-test). Scale bars, 200 μm. Figure **(D)** created with BioRender.com.

### 2.3 Cell-type-specific responses to IFNg treatment reveal CAF phenotypic transition to apCAFs in assembloids

Although IFNg is a well-characterized cytokine, less is known about its effects on specific cell types. To this aim we subjected untreated and IFNg-treated assembloids to single-cell RNA-sequencing (scRNA-seq) (Figure 3A). First, expression analysis of the IFNg receptors (IFNgR1 and IFNgR2) confirmed broad expression across the different assembloid compartments, with the highest enrichment observed in macrophages, fibroblasts, dendritic cells (DCs), and colorectal cancer cells across multiple patient lines (Figure 3B). Upon IFNg treatment, these specific clusters exhibited a robust upregulation of key T-cell attracting chemokines, namely, CXCL9, CXCL10, and CXCL11, with fibroblasts and macrophages showing the most prominent induction (Figure 3C). In parallel, IFNg stimulation strongly induced the expression of critical immune checkpoint and immunomodulatory molecules, including CD274 (PD-L1) and IDO1, primarily within the macrophage, fibroblast, DC, and colorectal cancer cell populations (Figure 3D), indicating a prominent role of the stroma in mediating immune-suppression upon IFNg treatment. Consistently, IFNg triggered an upregulation of MHC class I genes (HLA-A, HLA-B, and HLA-C), with the most pronounced increase in the fibroblast, myeloid and CRC cell compartments (Supplementary Figure S4). Quantification of both these pro- and anti-tumor transcriptomic changes (size effect) between the untreated and treated conditions identified fibroblasts as the most highly responsive cell population, followed closely by macrophages, DCs, and colorectal cancer cells (Figure 3E). To map the precise transcriptomic alterations driven by IFNg stimulation across distinct compartments in the assembloid model, we performed high-resolution differential gene expression analysis on isolated cell populations (Supplementary Figure S5). Analysis of the fibroblast compartment revealed a marked transcriptomic shift, characterized by the profound upregulation of classical interferon-stimulated genes including GBP5, GBP7, IDO1, IDO2, and CXCL9, alongside the downregulation of genes such as CCL26 and CCL11 (Supplementary Figures S5A,B). Similarly, the macrophage population exhibited extensive transcriptomic remodeling upon IFNg exposure. Differential expression highlighted a significant upregulation of genes such as CXCL11, RSAD2, LGALS17A, and SEPTIN4 (Supplementary Figures S5C,D). The epithelial compartment also showed a specific response, with IFNg-induced genes including CCL25, SERPING1, GBP5, TNFAIP6, and CXCL11 among the most significantly upregulated genes (Supplementary Figures S5E,F). Further analyses confirmed the robust induction of the T-cell attracting chemokines CXCL9, CXCL10, and CXCL11 (which was most prominent in the fibroblasts and macrophages) as well as the widespread upregulation of the immune checkpoint IDO1 and MHC-I molecules (HLA-A, HLA-B, HLA-C) across all three treated cell populations (Supplementary Figures S5B,D,F). Reactome pathway enrichment analysis further confirmed these functional shifts, showing highly significant activation of “Interferon-gamma signaling”, “PD-1 signaling”, and both class I and class II antigen presentation pathways across the fibroblast, macrophage, and CRC cell clusters (Figure 3F; Supplementary Figures S5G,H). Notably, analysis of the stromal compartment revealed that IFNg exposure drives a phenotypic transition within the fibroblast population. Module scoring demonstrated a distinct shift away from a classical myofibroblast (myCAF) baseline state toward an antigen-presenting CAF (apCAF) state (Elyada et al., 2019), that correlates to an IFNg response signature (IFNgCAF) (Figure 3G). This apCAF transition was characterized by a distinct gene signature highlighting the upregulation of apCAF markers, specifically MHC class II molecules (HLA-DRA, HLA-DRB1, CD74), alongside key transcription factors (IRF1, STAT1, and CIITA), and immune-modulatory genes (GBP1, IDO1, ICAM1) (Figure 3H). Conversely, structural myCAF markers such as COL1A1 and COL1A2 were downregulated in the treated condition (Figure 3H). To confirm this transcriptomic shift at the protein level, multiplex immunofluorescence staining for Vimentin and HLA-DR (MHC-II) validated a consistent increase in the proportion of Vimentin^+^ HLA-DR^+^ apCAFs within the total CAF population following IFNg exposure (Supplementary Figure S6A). Collectively, these single-cell profiles highlight how IFNg drives shared immunological features, while simultaneously triggering distinct, cell-type-specific transcriptomic changes across the tumor, stromal, and immune compartments, such as the remodeling of CAFs into an apCAF phenotype.

**FIGURE 3:**
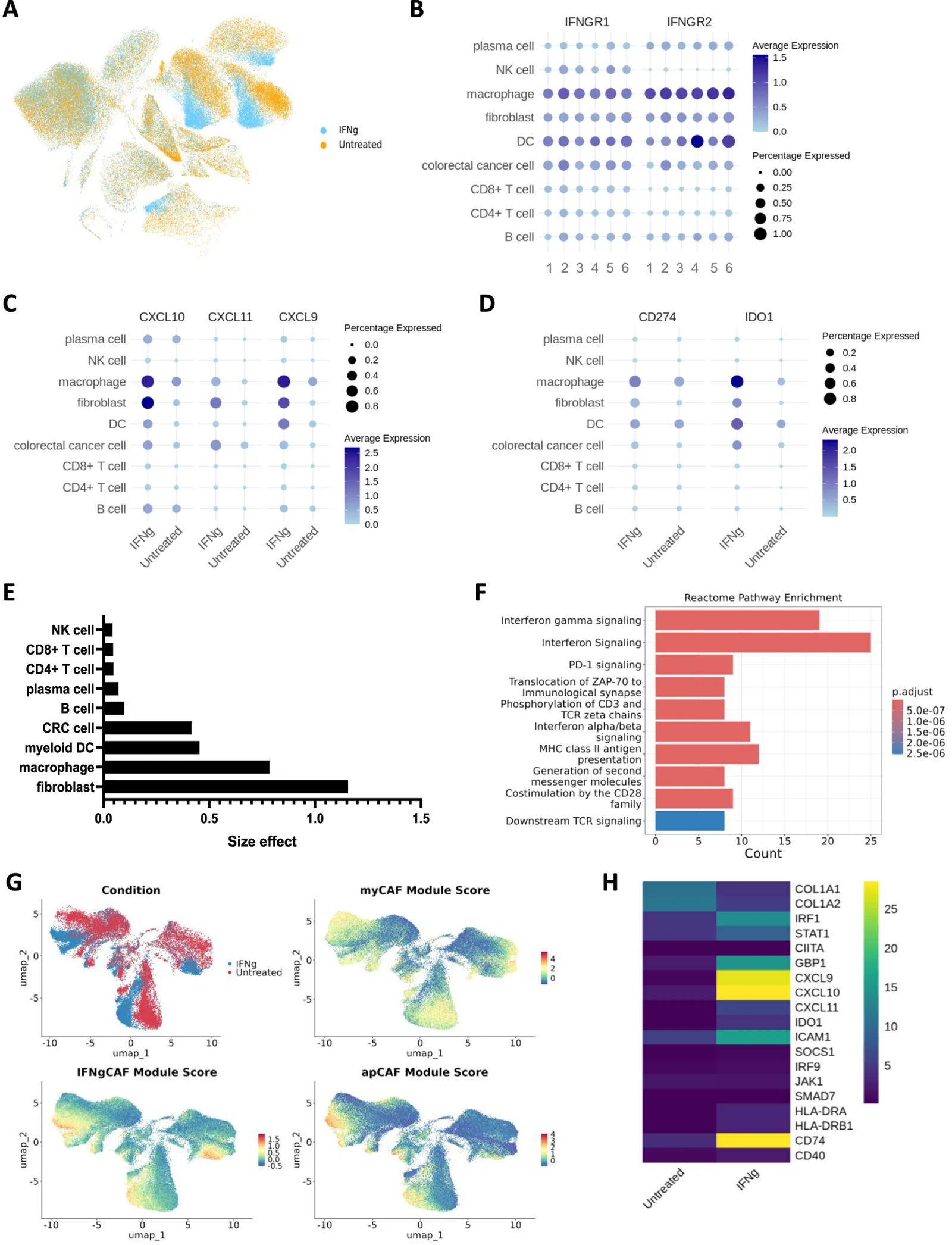
Cell-type-specific responses to IFNg treatment reveal CAF phenotypic transition to apCAFs in assembloids. **(A)** UMAP showing cell clusters under untreated and IFNg-treated (40 nM) conditions, with color-coded treatment groups. Treatment with IFNg results in distinct transcriptional changes in specific cell populations. **(B)** Dot plot displaying the expression of IFNGR1 and IFNGR2 across cell types, highlighting the presence of IFNg receptors in various populations. **(C)** Dot plot showing the expression of chemokines in response to IFNg treatment across cell types. **(D)** Dot plot illustrating the expression of immune suppressive factors in different cell types following IFNg treatment. **(E)** Bar plot quantifying the size effect of IFNg treatment on gene expression changes across cell types, with fibroblasts showing the largest response. **(F)** Reactome pathway enrichment analysis in fibroblasts reveals activation of pathways associated with interferon signaling, antigen presentation, and immune response regulation. **(G)** UMAP feature plots showing module scores myCAF, ifngCAF, and apCAF signatures across fibroblasts, demonstrating functional changes in fibroblast subsets. **(H)** Heatmap illustrating the expression of key interferon response genes in untreated and IFNg-treated assembloids.

### 2.4 Evaluation of novel FAP-targeting molecules in assembloids

Given the relevance of IFNg as a potent immunotherapeutic, its clinical application has historically been severely limited by peripheral dose-limiting toxicities and the induction of broad immunosuppressive feedback loops (Jorgovanovic et al., 2020; Gocher et al., 2022). To harness its anti-tumor potential while mitigating these systemic effects, we designed a novel panel of attenuated, targeted bispecific molecules intended to localize IFNgR activation specifically to the tumor stroma (Figures 4A,B). We utilized FAP, a marker predominantly overexpressed on CAFs, as tumor-enriching target to localize the cytokine’s activity. Immunohistochemical evaluation confirmed robust FAP expression within the fibroblast compartment of patient-derived assembloids, confirming their suitability for evaluating these stroma-targeted therapies (Figure 4C). To explore this, we engineered a panel of constructs with varying FAP binders and VHHs that bind IFNgR1 or IFNgR2 (Supplementary Figures S7A-E). Because these VHH molecules were specifically designed to be attenuated compared to the wild-type cytokine, we generated an untargeted reference molecule containing only the IFNgR-binding VHH domain, omitting the FAP-targeting arm, to serve as a reference. Alongside this, we engineered FAP-targeted molecules utilizing a two-molecule approach. In evaluating these constructs, we observed that they can exhibit distinct spatial mechanisms of receptor activation: cis activity, where the molecule binds to FAP and activates the IFNgR on the surface of the same cell (i.e., the CAF itself), and trans activity, where the molecule anchors to FAP on a CAF but bridges across to activate IFNgR on a neighboring cell. Additionally, we developed an “all-in-one” construct that integrates multiple anti-FAP binders (489, 212) and anti-IFNgR VHHs (R1, R2) into a single unified format capable of engaging both targets simultaneously. Optimization of the VHH chain fusion formats in 2D assays demonstrated that the physical geometry of these molecules strictly governs their functional activity. By fusing the VHH domains to either the light chain (LC) or heavy chain (HC) of the constructs, we found that structural configuration significantly impacted the molecules' IFNg signaling activation capacity. We quantitatively assessed this capacity by measuring shifts in average MHC-I expression levels (Figure 4D). We next evaluated the functional efficacy of these targeted IFNgR agonists within the complex 3D assembloid model. As expected, dose-response evaluations revealed that both recombinant IFNg and the non-targeted reference molecule potently induced CXCL10 secretion (Supplementary Figure S7F). Importantly, the targeted cis and trans combination treatments, despite being appropriately attenuated compared to the untargeted recombinant IFNg, still successfully induced CXCL10 secretion, demonstrating their therapeutic potential (Supplementary Figure S7F). Based on this dose-response profile, a concentration of 100 nM was selected for subsequent single-dose comparative experiments to assess the molecules at an effective concentration. We further investigated how this attenuated design translated to the broader tissue phenotype.

**FIGURE 4:**
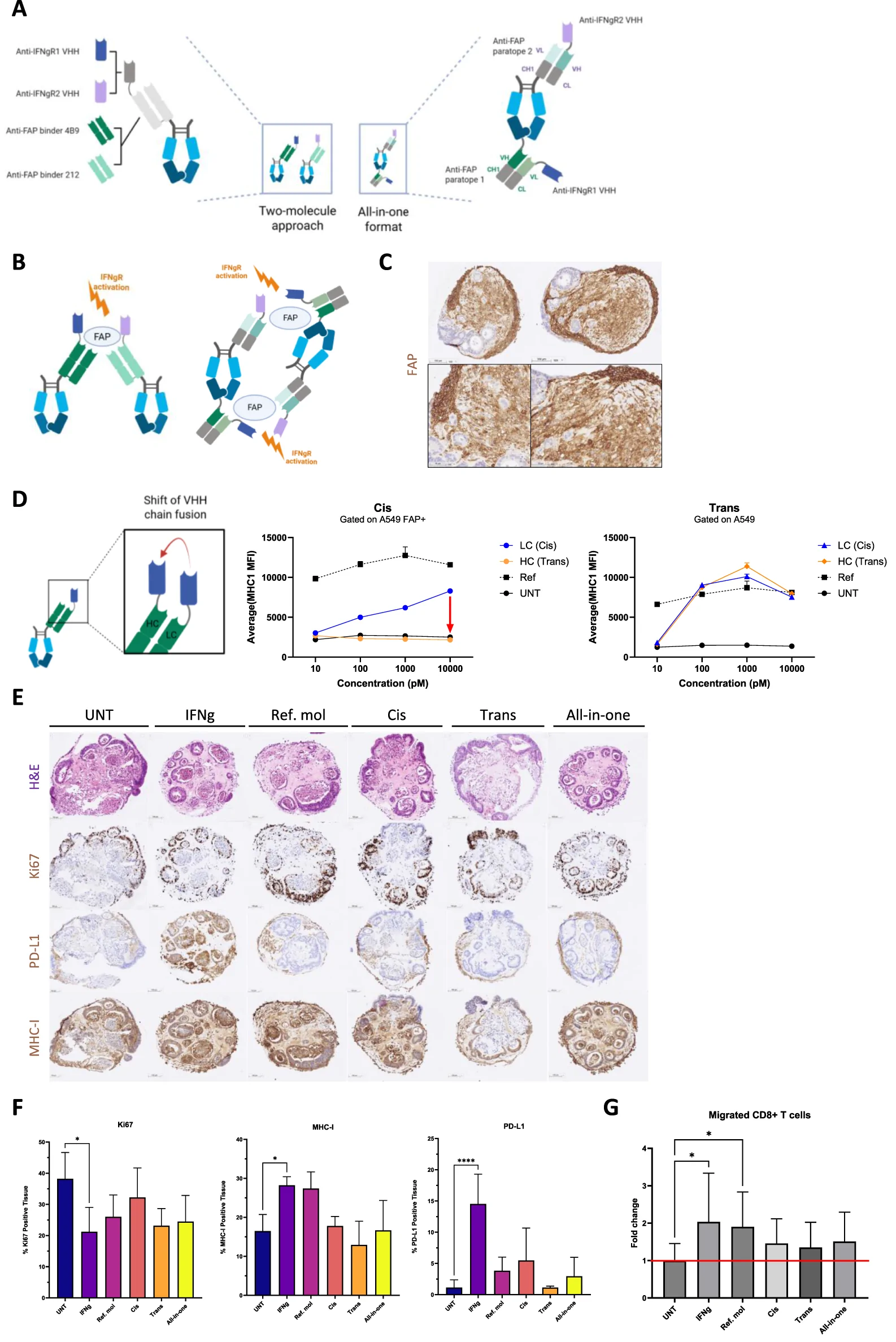
Development and evaluation of novel FAP-targeting molecules in assembloids. **(A)** Schematic of two FAP-targeting strategies: the two-molecule approach (separate FAP binder and anti-IFNgR1 or anti-IFNgR2 VHH) and the all-in-one format (containing both IFNgR VHHS). **(B)** Illustration of the mode of action of the FAP-targeting construct binding to FAP-expressing cells and subsequent IFNgR activation. **(C)** Immunohistochemistry showing FAP expression in the assembloids. Scale bars, 100 μm. **(D)** Schematic of VHH chain fusion shift and quantification of binding affinity in cis and trans configurations (Left). Graphs show dose-dependent MFI (mean fluorescence intensity) values for MHC-I for each treatment (cis and trans combination, reference molecule and untreated control) (Right). **(E)** Representative H&E and immunohistochemical staining of assembloids treated with IFNg, reference molecule, cis, trans, and all-in-one FAP-IFNg molecules, all in 100 nM. Scale bars, 100 μm. **(F)** Quantification of Ki67, MHC-I, and PD-L1 staining in assembloids, highlighting differences in immune marker expression and proliferative activity upon treatment. Data are presented as mean ± SD (n = 3 or 4 independent patients (biological replicates), one-way ANOVA with Dunnett’s multiple comparison test). **(G)** Transwell migration assay showing enhanced CD8+ T-cell recruitment in response to FAP-IFNg-treated assembloids. Data represented as fold change, normalized to baseline migration (red line). Fold change of the ratio of cell count, normalized to bead counts. Data are presented as mean ± SD (n = 3 independent patients (biological replicates), one-way ANOVA with Tukey’s multiple comparison test). Figure A, B and left panel of D created with BioRender.com.

Histological analysis confirmed that while recombinant IFNg significantly reduced the percentage of proliferative Ki67-positive tissue, the targeted (cis/trans) molecules exhibited a clear trend toward reduced Ki67 levels compared to the untreated group, though formal statistical comparisons did not reach significance (Figure 4E,F). Crucially, we observed that while standard IFNg treatment resulted in a massive upregulation of both MHC-I and the immunosuppressive checkpoint PD-L1, the cis, trans, and all-in-one targeted molecules elicited minimal PD-L1 upregulation (Figure 4E,F). Similarly, transwell migration assays showed a trend toward enhanced CD8^+^ T-cell recruitment with FAP-IFNg treatment, however, these changes did not achieve statistical significance over untreated controls, reflecting modest pro-migratory efficacy at the tested dose compared to the robust, statistically significant response driven by recombinant IFNg and the reference molecule (Figure 4G). To address whether this chemotactic recruitment coincided with immune cell stimulation, we orthogonally evaluated early T-cell activation via cell-surface CD69 expression in 2D co-cultures. Flow cytometric analysis revealed that exposure to IFNg variants induced dose-dependent upregulation of CD69 on both CD4^+^ and CD8^+^ T cells, providing evidence of early immune activation alongside chemotactic signaling (Supplementary Figure S7G).

Collectively, these findings demonstrate that our 3D assembloid platform is highly capable of capturing the complex, nuanced biology of engineered immunotherapeutics. The FAP-targeted constructs faithfully exhibited the attenuated effects they were designed for, delivering localized therapeutic activity such as chemokine secretion, anti-proliferative trends, and T-cell recruitment, while successfully avoiding the severe PD-L1 immunosuppressive feedback loop typically driven by broad IFNg exposure. Ultimately, this confirms that patient-derived assembloids provide a robust, physiologically relevant model to assess complex cellular interactions and screen the localized efficacy of novel, next-generation immunotherapies.

## 3 Discussion

Cancer immunotherapy has transformed the oncology landscape, yet clinical success remains highly limited. CRC is a strong example of this therapeutic gap, highlighting the urgent need for next-generation immunotherapies. The successful development of these immunological therapies is frequently hindered by a lack of advanced preclinical models that accurately reflect the human TME and that could mechanistically evaluate therapeutic responses. In this study, we applied and validated an established 3D patient-derived tumor-immune assembloid model integrating CRC organoids, CAFs, and immune cells to evaluate responses to IFNg and to a novel class of immuno-biologics aiming at delivering IFNg specifically to the TME. This platform serves as a robust translational tool that faithfully recapitulates the spatial architecture and cellular heterogeneity of the TME, enabling the dissection of cell-type-specific responses and complex drug modes of action.

The successful application of our 3D platform builds strongly upon the foundational shaking organoid co-culture (SHOCO) model recently established by Filip et al. (2026). In their work, Filip and colleagues demonstrated that this model faithfully captures the dense, fibrotic architecture of the human CRC microenvironment, preserving functional immune interactions and diverse cellular populations, including myeloid cells, far better than conventional co-cultures. While their study highlighted how this dense stromal network acts as a physical barrier restricting T-cell infiltration during immunotherapy with T cell engagers, our findings expand on this by revealing the profound dynamic plasticity of this barrier. We show that IFNg exposure remodels these structurally restrictive CAFs into an antigen-presenting phenotype (apCAF). Furthermore, Filip et al. showed that this stromal compartment can be exploited therapeutically using FAP-targeted co-stimulatory molecules (such as FAP-4-1BBL) (Filip et al., 2026). This study advances this therapeutic framework by characterizing FAP-targeted IFNgR agonists and delineating the functional differences between their cis and trans spatial engagement formats. Crucially, our findings underscore the immense value of this assembloid platform as a powerful tool to screen novel compounds and deeply investigate their complex mechanisms of action within the tumor stroma.

A central focus of our study was to map the localized biological responses to IFNg. As extensively highlighted in recent literature, IFNg acts as a “double-edged sword”, serving as both a teammate and an opponent within the TME (Gocher et al., 2022; Liu, 2025). On one hand, IFNg is a potent driver of anti-tumor immunity that induces tumor cell cycle arrest, enhances antigen presentation, and promotes the secretion of key chemokines (CXCL9, CXCL10, CXCL11) to recruit cytotoxic T cells (Gocher et al., 2022; Kureshi and Dougan, 2025; Liu, 2025). On the other hand, chronic exposure to IFNg triggers adaptive immune resistance by inducing immunosuppressive feedback loops, most notably through the robust upregulation of immune checkpoint PD-L1 and IDO1 (Ivashkiv, 2018; Gocher et al., 2022; Liu, 2025). Our patient-derived assembloids faithfully captured this multifaceted paradox. Treatment with IFNg resulted in macroscopic growth suppression, reduced Ki67 proliferation, and significantly enhanced CD8^+^ T-cell migration, while simultaneously driving a widespread upregulation of MHC-I and the immunosuppressive checkpoint PD-L1. While the transcriptomic readouts confirm the robust execution of canonical STAT1/IRF1 target programs across primary patient lines, we acknowledge that these data do not constitute direct biochemical characterization of proximal IFNGR–JAK–STAT phosphorylation kinetics within assembloids. Notably, when the allogeneic PBMC pool was held constant across experiments, the observed inter-patient variability in chemokine secretion primarily reflects intrinsic differences in tumor-stroma sensitivity to IFNg, though potential background alloreactive responses remain a recognized confounding variable in non-autologous settings. Crucially, while IFNg responses are typically studied in immune or tumor epithelial cells, our study provides biological insights into stromal plasticity by identifying the stromal fibroblast compartment as the most highly responsive cell population to IFNg stimulation. We uncovered that IFNg exposure drives a phenotypic transition in these cells, shifting them away from a classical structural myofibroblast (myCAF) phenotype toward an antigen-presenting CAF (apCAF) state.

This apCAF transition is characterized by the robust expression of MHC class II molecules (such as HLA-DR, HLA-DRA, and CD74). While direct antigen-specific presentation assays in primary human 3D models remain logistically challenging due to the need for matched autologous TCR systems, the functional capacity of MHC-II^+^ apCAFs to process antigens and directly modulate T cells is well established in prior literature. Landmark studies by Elyada et al., Kerdidani et al., and Huang et al. demonstrated that MHC-II^+^ CAFs act as functional non-professional antigen-presenting cells capable of binding antigens and directly interacting with T cells to regulate intratumoral immune activation (Elyada et al., 2019; Huang et al., 2022; Kerdidani et al., 2022). Our findings demonstrate that 3D patient-derived assembloids faithfully capture this stromal transition toward an antigen-presenting state upon IFNg pathway activation.

Historically, the clinical applicability of recombinant IFNg therapies has been largely unsuccessful due to a short half-life and severe, dose-limiting systemic toxicities (Gocher et al., 2022; Vorona et al., 2025). However, IFNg-based therapies have already achieved clinical success in certain hematological malignancies. For instance, Actimmune (recombinant human IFNg1b) combined with donor lymphocyte infusion safely enhances the graft-versus-leukemia effect, driving complete remission in patients with relapsed acute myelogenous leukemia and myelodysplastic syndrome following allogeneic hematopoietic stem cell transplantation (Vorona et al., 2025). Additionally, localized delivery of the human IFNg gene via the TG1042 adenovirus has yielded highly beneficial clinical responses in patients with cutaneous T-cell lymphoma (Gocher et al., 2022). Despite these encouraging results in blood cancers, broad systemic administration of IFNg paradoxically triggers adaptive immune resistance by upregulating immunosuppressive molecules such as PD-L1 and IDO1 within the tumor microenvironment (Jorgovanovic et al., 2020; Gocher et al., 2022). To counteract these detrimental feedback loops and safely concentrate the effects of IFNg at the tumor site, modern clinical research has evolved. Current strategies increasingly focus on combining IFNg with immune checkpoint inhibitors (ICIs) to overcome PD-L1 upregulation (Jorgovanovic et al., 2020; Gocher et al., 2022; Vorona et al., 2025). Furthermore, the field is advancing localized delivery systems, such as genetically modified cell therapies and viral vectors. To harness the potent immunostimulatory power of IFNg while circumventing its systemic adverse effects and broad immunosuppression, researchers are moving toward targeted cytokine delivery (Gocher et al., 2022; Kureshi and Dougan, 2025). Here, we evaluated a novel panel of engineered bispecific IFNg receptor agonists designed to anchor to FAP, thereby safely sequestering downstream IFNg activity specifically within the CAF-rich tumor stroma.

Our functional evaluation within the 3D assembloids demonstrated that localizing IFNg activity via FAP successfully uncouples the beneficial anti-tumor mechanisms (such as MHC-I upregulation) from detrimental immunosuppressive feedback loops. While standard recombinant IFNg provoked a great induction of CXCL10 secretion, it also drove widespread PD-L1 upregulation. In contrast, the FAP-targeted cis and trans constructs significantly attenuated chemokine release to fine-tuned levels. At the same time, these targeted molecules elicited minimal PD-L1 upregulation compared to the unattenuated treatment, successfully avoiding the severe immunosuppressive feedback loop typically driven by a lack of attenuation. Importantly, despite the attenuated CXCL10 secretion observed with the FAP-targeted constructs, the transwell migration assays showed a moderate increase in CD8^+^ T-cell recruitment, consistent with retention of some chemotactic activity. Nevertheless, chemotactic migration alone does not establish full cytotoxic T-cell activation. Evaluating downstream T-cell effector functions, such as granzyme B/perforin secretion, CD69/CD25 upregulation, and direct tumor cell cytolysis, was beyond the scope of this recruitment model, but remains an essential next step for fully autologous systems or *in vivo* studies. Overall, the attenuation of these IFNg molecules successfully impairs undesirable downstream effects such as PD-L1 induction, while retaining measurable positive features of IFNg pathway activation. However, the weaker effects observed for other functional results, including proliferation and T-cell recruitment, indicate that the current data do not establish whether the degree of attenuation achieved by these constructs provides an optimal pharmacodynamics balance. Rather than relying on simple concentration adjustments, future structural and molecular engineering should therefore focus on maximizing target-dependent IFNgR activation while maintaining limited target-independent signaling. Specific structural modifications could include tuning the binding affinity of the anti-IFNgR VHH domains, altering domain stoichiometry, such as employing bivalent VHH formats, or adjusting the length and flexibility of the inter-domain linkers to optimize the spatial geometry required for efficient receptor engagement upon FAP binding.

Despite its strengths, our study has some limitations. The assembloid model, while complex, still lacks a functional vasculature, which would influence both immune cell trafficking and drug delivery (Polak et al., 2024). Furthermore, the use of allogeneic PBMCs can introduce some baseline immune reactivity. Future iterations could incorporate autologous immune cells and endothelial networks to further enhance the model’s physiological relevance. Future work should also focus on optimizing the molecular design of the FAP-IFNg constructs to improve activation efficiency upon target engagement without compromising their attenuated profile.

In conclusion, this study applies and validates a highly relevant, patient-derived assembloid platform for the preclinical evaluation of advanced immunotherapies. We used it to dissect the cell-type-specific biology of IFNg, specifically uncovering apCAF transition dynamics, and provide a proof-of-concept characterization for a FAP-targeted cytokine delivery strategy. Importantly, the utility of this platform extends far beyond IFNg; by faithfully capturing the multicellular crosstalk of the human TME, this model is broadly applicable for studying the complex modes of action of other cytokines and emerging immunomodulators. This work paves the way for a more refined, human-derived *in vitro* strategy to complement translational research, accelerating the discovery and optimization of next-generation, targeted immunotherapies for solid tumors.

## 4 Limitations of the study

While the 3D immune-containing model provides a robust platform for studying tumor-immune interactions and therapeutic responses, several limitations should be acknowledged. First, the model relies on *in vitro* conditions that may not fully replicate the complexity of the *in vivo* tumor microenvironment, including factors such as vascularization, real-time immune trafficking, and systemic interactions. Second, the observed patient-specific differences in responses to IFNg highlight the inherent variability of primary cells, which can pose challenges in standardizing experimental outcomes. Finally, while the model successfully captures key aspects of IFNg signaling, further validation is required to assess its ability to recapitulate other cytokine-driven or therapy-induced changes. Addressing these limitations in future iterations of the model could enhance its applicability to translational research and personalized medicine.

## 5 Materials and methods

### 5.1 Cell isolation

#### 5.1.1 Organoids and fibroblast sourcing

Colorectal cancer organoids, cancer-associated fibroblasts (CAFs), and associated clinical information were derived from tissues obtained from patients undergoing tumor resection at the University Center for Gastrointestinal and Liver Disease (Clarunis), Basel, Switzerland. The organoids and fibroblasts were sourced through a collaborative framework adhering to legal and ethical regulations. The project was performed in accordance with the Helsinki Declaration and was reviewed and approved by the Ethics Committee of Basel (EKBB, no. 2019–02118).

#### 5.1.2 Colorectal cancer tissue digestion

Colorectal cancer tissues were washed five times with PBS supplemented with penicillin-streptomycin (Thermo, 15070063), gentamicin (Thermo, 15750060), and amphotericin (Thermo, J67049-AD). Clean tissue was minced and enzymatically digested in 1 mL of advanced DMEM/F-12 (GIBCO, 12634028) medium supplemented with 20 U/mL and 20 μg/mL of P/S (ThermoFisher Scientific, 15140122), Primocin 0.1 mg/mL (InvivoGen, ant-pm-1), Hepes 10 mM (GIBCO, 15630056), 2 nM Glutamax (GIBCO, 35050061), 4 mg/mL collagenase IV (Worthington, LS004189), 0.1 mg/mL DNase IV (Sigma, D5025), 20 μg/mL hyaluronidase V (Sigma, H6254), 1% BSA (Sigma, A3059), and 10 μM LY27632 (Abmole Bioscience, M1817) for 1 h at 37 °C under slow rotation, with vigorous pipetting every 15 min. The tissue lysate was filtered through a 100 μM cell strainer and centrifuged at 300 *g* for 10 min. The cell pellet was then resuspended in advanced DMEM/F-12 and the cells were counted.

#### 5.1.3 CAF culture establishment

For CAF culture establishment, 1–2 × 10^6 cells were plated in a 6-well plate with Mesenchymal Stem Cell Growth Medium 2 (Promocell, C-28009). The medium was replaced every 2–3 days, and cells were split using Trypsin 0.05% (Gibco, 25300054) when confluent. CAF cultures were maintained in a humidified incubator at 37 °C with 5% CO2 and atmospheric O2 (21%).

#### 5.1.4 Colorectal cancer organoid culture establishment and passaging

For organoid culture establishment, 1.6 × 10^5 cells were seeded in 20 μL Matrigel domes (Corning, 356,231) in a culture plate. Plates were incubated inverted at 37 °C for 30 min before the addition of organoid media, prepared as previously described by Sato et al. (2011). Media was supplemented with 1 uM Y-27632 (Rock-Inhibitor, Bio-Techne, 1254) after seeding. Organoid cultures were maintained in a humidified incubator at 37 °C with 5% CO2 and atmospheric O2 (21%). The media was refreshed every 48–72 h.

For passaging, typically every 7 days, domes were dissociated using Gentle Cell Dissociation Reagent (StemCell Technologies, 100–0485). Following a 10-min incubation with periodic mechanical disruption, the suspension was centrifuged at 250 *g* for 4 min and washed with ice-cold Advanced DMEM-F12 containing 1% BSA (A9576, Sigma-Aldrich) and HEPES (Gibco, 15,630–056) to ensure complete removal of the hydrogel. Isolated PDOs were resuspended in fresh Matrigel and replated. Plates were incubated inverted at 37 °C for 20 min before the addition of culture media supplemented with 1 uM Y-27632 (Rock-Inhibitor, Bio-Techne, 1254). Culture media consisted of a 1:1 mixture of Advanced DMEM-F12 (Gibco, 12,634–010) + GlutaMax-I (Gibco, 35,050–038) + Penicilin/Streptomycin (P/S) (Gibco, 15,140–122) and supplemented OGM (StemCell, 100–0190). Organoid cultures were maintained in a humidified incubator at 37 °C with 5% CO2 and atmospheric O2 (21%).

#### 5.1.5 Isolation of peripheral blood mononuclear cells (PBMCs), freezing and thawing

Human peripheral blood mononuclear cells (PBMCs) were isolated from lymphocyte-enriched human blood preparations (buffy coats) using SepMate™-50 gradient separation, following the manufacturer’s instructions (StemCell, 85,450). CD8+T cells were further isolated using the CD8^+^ T cell isolation kit according to the manufacturer’s instructions (Miltenyi, 130–096–495). Buffy coats were obtained from the Zurich Blood Donation Center in compliance with the Declaration of Helsinki.

PBMCs were aliquoted at varying concentrations and cryopreserved in Cellbanker 1 (Amsbio, 11,910) for long-term storage in liquid nitrogen. To recover the cells for assembloid generation, cryovials were rapidly thawed in a 37 °C water bath for approximately 1 min. Once a minimal ice crystal remained, the cell suspension was gently homogenized and transferred dropwise into 10 mL of pre-warmed washing media (Advanced DMEM-F12 supplemented with P/S, HEPES, and 1% BSA) to minimize osmotic shock. The suspension was centrifuged at 230 *g* for 5 min), the supernatant aspirated, and the pellet subjected to an additional wash with 10 mL of washing media. Following a final centrifugation step, the PBMCs were resuspended in 5 mL of media and quantified for experimental use.

### 5.2 Assembloid assays

#### 5.2.1 Generation and culture of assembloids

Assembloids were formed as previously described (Filip et al., 2026). CRC organoids and CAFs were harvested, and PBMCs were thawed following as described before. A single 25 µL CRC organoid dome was used to prepare a 100 µL assembloid volume. This included 30,000 PBMCs/µl and 5,000 CAFs/µl, which were mixed with the organoids. 100 uL volume generated 20 assembloids, each with a final volume of 5 µL per well. For the scaling experiments, we used additional volumes of 10 and 20 µL per well. The cell suspension was centrifuged at 300 *g* for 5 min, and the supernatant was carefully removed to avoid disturbing the pellet. The cells were then resuspended in 2 mg/mL Collagen I (BIOMATRIX, 5225-50 ML) and maintained on ice until seeding.

For seeding, 100 µL of dOil (HFE3500, 3 M) mixed with 0.5% surfactant (dSURF, Fluigent) was added to each well of an ultra-low attachment U-bottom 96-well plate (SBio, MS-9096UZ). Five microliters of the cell suspension in collagen hydrogel were pipetted into each well, allowing the hydrogel to form a spherical structure and float beneath the oil surface. The plate was placed on a shaker at 150 rpm in an incubator for 20 min to allow for gel polymerization. After polymerization, the oil was carefully removed and pooled for reuse, while the plate was left open under the biosafety hood to evaporate residual oil. Finally, 100 µL of assembloid medium (a 1:1 mix of organoid and CAF medium) was added to each well, and the plate was cultured on a shaker at 150 rpm at 37 °C in a humidified incubator. The medium was refreshed every 2–3 days.

#### 5.2.2 *In vitro* drug treatments and downstream processing

Assembloid cultures were maintained for 72 h to allow for self-organization. Following this period, the media was replaced with media containing the experimental compounds, marking the start of treatment (day 3 of culture). Time points of interest were set at days 1, 3, 5 or 6. At each time point, the media was completely removed from the corresponding samples and stored at −20 °C for subsequent cytokine analysis.

#### 5.2.3 Fixation and embedding of assembloids

Assembloids were washed once with 100 µL of DPBS per well and transferred to a new 96-well plate containing 100 µL of cytofix fixation buffer (BD Biosciences, 554,655) per well for overnight fixation at 4 °C. On the following day, the PFA was removed, and the samples were washed twice with 100 µL of DPBS per well. To prevent drying, 100 µL of DPBS was added to each well before sealing the plate. The fixed samples were stored at 4 °C in the refrigerator until further use.

Histogel (Epredia, HG-4000–012) was liquefied in the microwave for 30 s and poured into a tissue microarray mold designed to hold 24 samples. The mold was filled completely and placed in the fridge at 4 °C for 15 min to allow the Histogel to solidify. Once solidified, the Histogel block was carefully removed from the mold and inverted, exposing cavities designed for the organized embedding of multiple samples. Fixed assembloids were individually placed into each cavity, and 10 µL of liquid Histogel was gently added on top of each assembloid to secure it to the block. The block was then left to dry at room temperature for 1 min before being completely filled with liquid Histogel, fully enclosing the assembloids. The block was returned to the fridge for 15 min to solidify. Once fully solid, the block containing the assembloids was placed into a plastic cassette and transferred into DPBS in preparation for dehydration and paraffin embedding, which were performed the next day.

The dehydration protocol was carried out using a tissue processor (Tissue-Tek VIP - 6 AI, Sakura). Samples were first treated with 4% Formalin for 30 min, followed by sequential treatments with increasing concentrations of ethanol: 70% ethanol (30 min and 1 h), 80% ethanol (30 min), 96% ethanol (30 min and 1 h), and 100% ethanol (1 h and 45 min). Ethanol-treated samples were then processed with xylene twice (45 min each). Finally, the samples were treated with paraffin three times (45 min each) at 56 °C. The following day, the samples were embedded in liquid paraffin using metallic molds and secured to the initial cassette printed using a laser printer (FA-TECH VEGA, Vega Laser Cassettes Marker), which served as an anchoring point for the microtome. The formalin-fixed paraffin-embedded (FFPE) blocks were sectioned (Epredia, HM355 S) at a thickness of 3 µm and transferred onto SuperFrost Plus Adhesion slides (Epredia, J1830AMNZ). Slides were dried overnight at room temperature.

### 5.3 Histology and imaging

#### 5.3.1 H&E and immunohistochemistry staining

VENTANA HE 600 system was used to perform H&E staining to assess tissue area and architectural morphology. FFPE tissue sections were stained with Ki67 (Cell Marque, 275R-16; Dilution 1:100), FAP (Abcam, ab240989; Dilution: 1/100), MHC-I (Abcam, ab70328; Dilution: 1/12,000), and PD-L1 (Abcam, ab228463; Dilution: 1/50), using the Leica Bond RX (Leica Biosystems, 21.2821). Tissue slides were processed using a standardized immunohistochemistry protocol with all reagents supplied by Leica Microsystems and dispensed at 150 µL per slide at ambient temperature. Slides were incubated with marker for 30 min, followed by three sequential bond wash rinses. Polymer was applied for 15 min, then two 2-min bond wash steps and one additional wash. Endogenous peroxidase was blocked with a 5-min peroxide block treatment, succeeded by two bond wash steps (the second using open dispense) and a final wash. Chromogenic visualization was achieved with mixed DAB refine (0 min initial incubation, then 10 min development), followed by three deionized water rinses. Hematoxylin counterstaining was performed for 5 min, then completed with sequential rinses of deionized water and bond wash solution. All incubations were conducted at ambient temperature. Slides were coverslipped using a Coverslipper (Medite, RCM7000) and mounted with pertex Mounting Medium (Medite, 41–4011–0).

#### 5.3.2 Multiplex IF staining

Tissue slides were prepared and stained using a sequential multiplexed immunofluorescence approach on a Leica Bond automated stainer, with all reagents supplied by Leica Microsystems. Briefly, slides were dewaxed and baked using Leica Dewax Solution, followed by three alcohol dispensions and three Bond Wash Solution rinses. Antigen retrieval was performed with three applications of Epitop Retrieval Solution, followed by five Bond Wash Solution rinses and one application of PKI Blocking Buffer. For each of six antibody-fluorophore pairs, the following steps were repeated: Primary antibodies (Ki67 (Cell Marque, 275R-16; Dilution 1:100), FAP (Abcam, ab240989; Dilution: 1/100), MHC-I (Abcam, ab70328; Dilution: 1/12,000), PD-L1 (Abcam, ab228463; Dilution: 1/50), E-Cadherin (Cell signaling, 3195; Dilution: 1/200), Vimentin (Abcam, ab16700; Dilution; 1/100), CD3 (Diagnostic BioSystems, RMBA005; Dilution: 1/100), CD4 (Abcam, ab133616; Dilution: 1/500), CD8 (Abcam, ab237709; Dilution: 1/1000), CD68 (Cell signaling, 76,437; Dilution: 1/100)) were applied, followed by three Bond Wash Solution rinses. Polymer HRP (Akoya Biosciences, ARH1001EA) was added, followed by five Bond Wash Solution rinses. The corresponding Opal dye (480 (Akoya Biosciences, FP1500001KT), 520 (Akoya Biosciences, FP1487001KT), 570 (Akoya Biosciences, FP1488001KT), 620 (Akoya Biosciences, FP1495001KT), 690 (Akoya Biosciences, FP1497001KT) or 780 (Akoya Biosciences, FP1501001KT)), was applied, followed by three Bond Wash Solution rinses. Between each antibody round, antigen retrieval was repeated with three Epitop Retrieval Solution applications, five Bond Wash Solution rinses, and one PKI Blocking Buffer (Akoya Biosciences, ARD1001EA) application prior to the next primary antibody incubation. Following the final Opal 780 dye application, slides were rinsed three times with Bond Wash Solution. DAPI (Akoya Biosciences, FP1490) was applied and followed by five wash steps with Bond Wash Solution. Slides were coverslipped using a CTM6 Coverslipper (Histocom AG) and mounted with Immu-Mount (Epredia, 9990414).

#### 5.3.3 Histological analysis

For brightfield applications, IHC-stained sections were scanned at a resolution of ×20 magnification utilizing the Olympus SLIDEVIEW VS200. Multiplexed immunofluorescence slides were acquired using the PhenoImager HT multispectral imaging system (Akoya Biosciences). Tissue area and percentage of positive cells were quantified using HALO imaging software (version 3.6).

#### 5.3.4 3D immunophenotyping (3DIP)

The fixed assembloids were embedded in low-gelling temperature agarose (Sigma-Aldrich, A0169) and sectioned into 70 µm thick slices using a vibratome (Leica VT1200 S) equipped with conventional razor blades. Sample sections were prepared for imaging as previously described (1). The sections were stained overnight using a mixture of the following antibodies: E-Cadherin (R&D, AF648, 1:400). Vimentin (Cell Signaling, 7675, 1:800), CD8 (BioLegend, 344,748, 1:200), CD4 (BD, 566,104, 1:200), Ki67 (Invitrogen, 58–5698–82, 1:600). The staining buffer consisted of 0.1 M Tris (Sigma-Aldrich, 10708976001) containing 0.3% Triton X (Sigma-Aldrich, X100), 1% mouse serum (Jackson ImmunoResearch, 015–000–120), and 1% bovine serum albumin (BSA, Sigma-Aldrich, A9576). Images were acquired using a Leica SP8 or Stellaris 5 inverted confocal microscope equipped with a ×40 objective lens, with a resolution of 512x512 pixels and a z-spacing of 2 µm. Image quantification, and visualization were performed using Imaris 10.1.1 (Bitplane).

### 5.4 Single-cell RNA-sequencing

#### 5.4.1 Sample preparation

Assembloids were enzymatically dissociated into single cell suspensions. Ten assembloids were pooled per condition in an Eppendorf tube. Assembloids were washed with PBS before adding 1 mL of pre-warmed digestion mix. The digestion mix contained 5 mg/mL collagenase II (Gibco, 17,101–015), 400 ug/mL Dnase I (Roche, 11284932001), 0.1% BSA (Sigma-Aldrich, A9576), 1 mg/mL Dispase II (Gibco, 17,105–041) and 20 uM Rock Inhibitor (Bio-Techne, 1254). The Eppendorf tube was placed in a water bath at 37 °C for 30 min. Every 5 min, the solution was pipetted up and down rigorously to break down the assembloid structure. After the incubation, the cells were washed with PBS and filtered through a 40 um cell strainer (Falcon, 352,340) to remove remaining aggregates. The cell number and viability were evaluated using a Nexcelom Cellometer Auto 2000 and a total of around 29,000 cells per sample were loaded into a 10x Genomics Chromium X instrument. Cell capture, cDNA and library preparation were performed according to the manufacturer’s indications (Chromium GEM-X Single Cell 5′Reagent Kits, v3). The concentration of the cDNAs and the final libraries was quantified using an Invitrogen Qubit fluorometer (High Sensitivity dsDNA Assay) and the respective fragment size distribution was determined using an Agilent 2100 Bioanalyzer automated electrophoresis instrument (High Sensitivity DNA kit). The libraries were pooled equimolecularly and sequenced in an Illumina NovaSeqX + sequencer according to 10x Genomics recommendations (R1 = 26, i7 = 10, i5 = 10, R2 = 90) to a depth of approximately 20,000 reads per cell for the GEX library.

#### 5.4.2 Data analysis

Raw sequencing reads were aligned to the human reference genome (GRCh38) via Cell Ranger Single Cell software v9.0.1 (10x Genomics) with the refdata-gex-GRCh38-2024-A reference package. Empty droplets were identified and removed from the unfiltered count matrices using Dropkick (v1.2.8) (Heiser et al., 2021), applying a minimum threshold of 200 genes. Doublet detection was performed via the scDblFinder R package (v1.12.0) (Germain et al., 2021) in R (v.4.4.1) prior to merging the per-sample raw expression matrices. Downstream analysis was implemented in Python (v3.9) utilizing Scanpy (v1.10.3). Low quality cells were excluded applying a dual-filtering strategy. First, an adaptive filter excluded barcodes deviating by more than three Median Absolute Deviations (MAD) from the median for: (1) total counts, (2) detected genes, and (3) mitochondrial gene fraction. Second, thresholds were applied to ensure the removal of low-quality remnants, retaining only cells with >500 genes and >1,000 counts. Following the dual filtering strategy cells with 500–7,323 genes, 1,000–31,943 counts, and <8.19% mitochondrial genes were retained in the final dataset. Subsequently, gene expression counts were transformed to log1p (cp10 k) values and scaled. Highly variable genes were identified and used as input for Principal Component Analysis (PCA). The first 50 principal components were utilized to construct a 10-nearest neighbor graph, which was further embedded into two-dimensional space using UMAP (Becht et al., 2019). Cell clusters were defined using the Leiden algorithm (Traag et al., 2019). Cell type assignments were performed with the semi-automated Sig-annot module within the besca package (Mädler et al., 2021). The gene sets used for these annotations are available at https://github.com/bedapub/besca/blob/main/besca/datasets/genesets/CellNames_scseqCMs6_sigs.gmt. Automated clusters were assigned at multiple levels of granularity, followed by manual refinement based on canonical marker gene expression. For differential expression analysis, cells were aggregated into pseudobulks by sample. To maintain statistical robustness, pseudobulk samples containing fewer than 10 cells were excluded from further analysis. This pseudobulk aggregation was implemented using the decoupler Python package (v1.9.0). Differential gene expression was then assessed on the unnormalized raw integer counts utilizing PyDESeq2 (v0.4.12). To account for patient-specific baseline variations, the patient identifier was included as a covariate in the design matrix alongside the experimental condition. P-values were adjusted for multiple testing via the Benjamini–Hochberg procedure, defining significantly differentially expressed genes by an adjusted p-value <0.05 and an absolute log2 fold change >0.5. Finally, to explore the functional implications of the transcriptional responses, Over-Representation Analysis (ORA) was conducted on the significantly upregulated genes utilizing the Enrichr module from the gseapy package (v1.1.11). Pathway enrichment was evaluated against the KEGG 2021 Human, GO Biological Process 2021, and Reactome 2022 databases, with significantly enriched pathways defined by an adjusted p-value <0.05.

### 5.5 Cytokine analysis

#### 5.5.1 ELISA

To evaluate the functional secretory phenotype of the assembloid models, conditioned media from untreated and IFNg-treated cultures were collected after the specified incubation periods and centrifuged to remove cellular debris. The secretion of the chemokine CXCL10 was quantified using R&D Systems DuoSet ELISA (DY266) per manufacturer’s instructions. Absorbance was measured using a microplate reader, and absolute concentrations were calculated against a standard curve.

#### 5.5.2 Multiplex

For broader inflammatory profiling, the supernatant of assembloids was collected and stored at −80 °C. Samples were thawed and cytokine content analyzed by Luminex technology using Bio-Plex Pro Human cytokine Panel 48-plex (Bio-Rad, 12007283) following manufacturer’s instructions. Sample acquisition and cytokine concentrations were determined using a Bio-Plex 200 System (Bio-Rad) and its integrated software. Figures were generated with Prism v10 (GraphPad Software).

### 5.6 Transwell migration assays

For naïve CD8^+^ T cell migration assays, two assembloids were placed in the lower chamber of a 96-well transwell plate. Transwell inserts with a 5 µm pore size (Corning, CLS3387-8 EA) were used, and 150 µL of assembloid medium, either without compounds or containing double the concentration of the compounds, was added to the assembloids. The plate was incubated for 4 h at 37 °C in a CO_2_ incubator.

Following incubation, CD8^+^ T cells were thawed and stained with 4 uM CellTracker green CMFDA (Invitrogen, C7025) for 10 min at 37 °C in the dark. After quenching the reaction with ice-cold FBS, cells were centrifuged at 250 *g* for 5 min. Cells were resuspended in assembloid medium and counted. Afterwards, a total of 150 µL of assembloid medium containing 500,000 labeled CD8^+^ T cells was added to the upper chamber of the transwell plate. The naïve CD8^+^ T cells were allowed to migrate for 48 h. Cells that migrated to the lower chamber were collected and quantified via flow cytometry. Absolute cell counts were normalized using 123count eBeads (Invitrogen, 01–1234–42) according to the manufacturer’s protocol. All the acquired data from the flow cytometry analyses were analyzed using FlowJo 10.10.0 (BD Biosciences).

### 5.7 Molecules

The FAP-IFNg molecules used in this study are IgG1-based constructs designed with bivalent binding entities targeting fibroblast activation protein (FAP) and monovalent binding to interferon-gamma receptor 1 or 2 (IFNgR1 or IFNgR2). These constructs feature a silent Fc region engineered with a P329G LALA mutation to prevent binding to FcγR, minimizing unintended Fc-mediated interactions. As a reference, a non-targeting IFNg molecule was generated with nonbinding entities in place of the FAP-targeting binders. All molecules were produced internally, using either HEK-293 T or CHO mammalian expression systems via transient transfection. Following expression, the proteins underwent a rigorous three-step purification protocol and subsequent sterilization. Quality oversight was maintained through liquid chromatography-mass spectrometry (LC-MS) for protein identification, analytical chromatography for purity assessment, and standardized assays for endotoxin quantification and concentration determination. The finalized constructs were formulated in a sterile buffer consisting of 20 mM Histidine and 140 mM NaCl (pH 6.0) and stored under standardized conditions until experimental use.

### 5.8 2D cell culture

#### 5.8.1 Maintenance

The human lung carcinoma cell lines A549_CEA were cultured in DMEM (Gibco, 31,966–021) supplemented with 10% (v/v) heat-inactivated fetal bovine serum (FBS), 1% penicillin-streptomycin, and 1% sodium pyruvate. The immortalized cancer-associated fibroblast line iHBCAF (donor 25,299, clone 04) was maintained in huCAF medium (Inscreenex, INS-ME-1036). Cells were maintained in a humidified atmosphere at 37 °C with 5% CO2. For routine passaging, cells were harvested at 70%–80% confluence using 0.25% Trypsin-EDTA (Gibco 25,300–054) for 5 min, followed by neutralization in complete growth medium. Cell viability and counts were assessed using a motorized cell counter prior to each experimental setup.

#### 5.8.2 Untargeted and CIS TRANS activation assay

For co-culture assays, A549_CEA cells (1–5x 10^6 cells) were harvested and washed twice with PBS. Cells were resuspended in 1 mL of 5 µM CellTrace™ Violet (CTV, Thermo Fisher Scientific, C34557) solution and incubated at 37 °C for 15 min. The labeling reaction was quenched by adding 10 mL of complete medium and incubating for an additional 5 min. Following two washes in complete medium, CTV-labeled A549_CEA cells were counted and seeded at a density of 7,000 cells per well in 96-well flat-bottom plates for the untargeted conditions. For co-culture conditions, A549_CEA huFAP cl48 cells and A549_CEA cells were added at a 1:1 ratio (3,500 cells/well) in 50 µL each. Plates were incubated overnight to allow for cell adherence. To evaluate the induction of MHC-I and PD-L1, cells were treated with molecules at assay specific concentrations (indicated in figure). Plates were incubated at 37 °C for 72 h. Following the incubation, the wells were washed. Cells were detached with 50 µL trypsin per well for 10 min at 37 °C. The reaction was stopped with 100 µL of complete medium, and the cell suspensions were transferred to a 96-well U-bottom plate.

#### 5.8.3 PBMC activation assay

Target cells (A549 WT and CAFs) were harvested using trypsin/EDTA, checked for viability, and centrifuged at 480 *g* for 4 min. Pelleted cells were resuspended at a density of 0.2 × 10^6 cells/mL in complete medium (DMEM supplemented with 10% FBS and 1% penicillin/streptomycin). Target cells were seeded into 96-well flat-bottom tissue culture plates at a final density of 20,000 cells/well (100 uL/well) of single target cell suspensions, or 50 uL CAFs and 50 uL A549 WT cells for co-culture conditions) and incubated overnight at 37° in a humidified incubator to allow for cell adherence. On Day 2, fresh PBMCs were resuspended in assay medium at 2 × 10^6 cells/mL. To each well, 50 uL of medium containing compound serial dilutions or controls was added, followed immediately by 50 uL of the PBMC cell suspension (1 × 10^5 PBMCs/well). Plates were incubated at 37° for 48 h. Following the incubation supernatants were taken, the wells were washed. Cells were detached with 50 µL trypsin per well for 10 min at 37 °C. The reaction was stopped with 100 µL of complete medium, and the cell suspensions were transferred to a 96-well U-bottom plate.

#### 5.8.4 Flow cytometric analysis

Cells were washed with PBS and incubated with human Fc block (1:50 dilution, BD Biosciences, 564,219) in PBS for 10 min to prevent non-specific binding. Subsequently, a master mix containing fixable Live/Dead dye (1:1000 dilution, Zombie Aqua, Biolegend or L10119, LD NIR, APC-Cy7 Thermo Fisher) and fluorophore-conjugated antibodies against MHC-I (1:400 dilution, Biolegend, 311,410, clone W6/32, APC fluorophore), PD-L1 (1:400 dilution, Biolegend, 329,714, clone 29 E.2A3, BV421 fluorophore), CD69 (1:200, BD, 560,967, clone FN50, APC fluorophore), CD3 (1:200, BD, UCHT1 563,423, PE-Cy7 fluorophore), CD4 (1:100, clone OKT4, Biolegend, 317,426, AF700 fluorophore), or CD8 (1:100 RPAT8, BD, 301,043, BV711 fluorophore was added. Staining was performed for 30 min at 4 °C in the dark. Cells were washed with FACS buffer (PBS +0.5% BSA) and fixed with 50 µL of BD Cytofix (4% PFA, BD Biosciences, 554,655) for 20 min at 4 °C. After two final washes in FACS buffer, cells were resuspended in 90 µL FACS buffer and analyzed using a BD LSRFortessa flow cytometer.

### 5.9 Statistical analysis

Statistical analyses were performed using GraphPad Prism software (version 10.3.1). Normal distribution was verified, and data were subsequently analyzed as described in the Figure legends. Data are presented as the mean ± Standard Deviation (SD). P-values ≤0.05 were considered statistically significant. Significance levels are denoted as follows: ns or not indicated (not significant) = P > 0.05; * = P ≤ 0.05; ** = P ≤ 0.01; *** = P ≤ 0.001; **** = P ≤ 0.0001.

## 6 Illustrations

Some illustrations were created with BioRender.com, as indicated in the figure legends.

## Data Availability

All data and protocols in this manuscript are available to share upon request to the corresponding authors.
