# Supplementary material for: Evaluating cytokine-based therapeutics in human 3D in vitro immune-containing assembloids
Source: Front Cell Dev Biol. 2026 Oct 15;14:1930651. doi: 10.3389/fcell.2026.1930651 (PMC13717148; doi:10.3389/fcell.2026.1930651)
Supplement: Supplementary file 1 [file Supplementaryfile1.pdf]

FIGURE 1

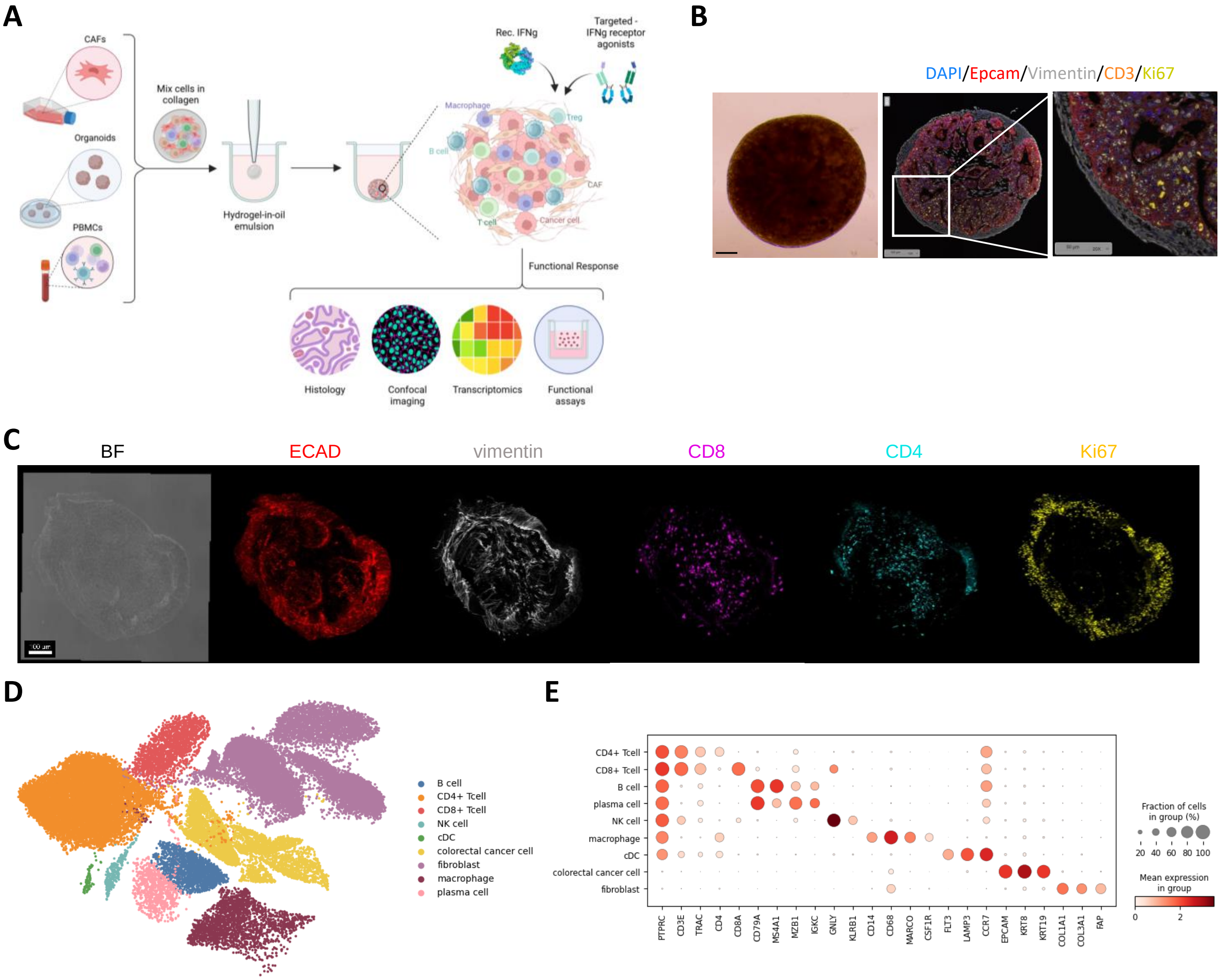

Fig. 1: Colorectal assembloid generation and characterization.
(A) Schematic of CRC assembloid generation. (B) Brightfield image of assembloids at day 6. Scale bar, 100 µm. Representative immunofluorescence staining of the assembloid showing DAPI (nuclei, blue), EpCAM (epithelial cells, red), Vimentin (fibroblasts, white), CD3 (T cells, yellow), and Ki67 (proliferation marker, yellow). Scale bars indicate 100 µm (left) and 50 µm (right). (C) Representative images of cellular composition and organization within the assembloid, showing BF (bright field), ECAD (red), Vimentin (grey), CD8 (purple), CD4 (cyan), and Ki67 (yellow). Scale bar: 100 µm. (D) UMAP visualization of single cell gene expression with cells colored by cell identity, aggregated from assembloids across n = 6 independent patients (biological replicates). (E) Dot plot showing marker gene expression for each cell cluster, confirming the identity of the distinct populations present in the assembloid. Panel (A) created with BioRender.com.

SUPPLEMENTARY FIGURE 1

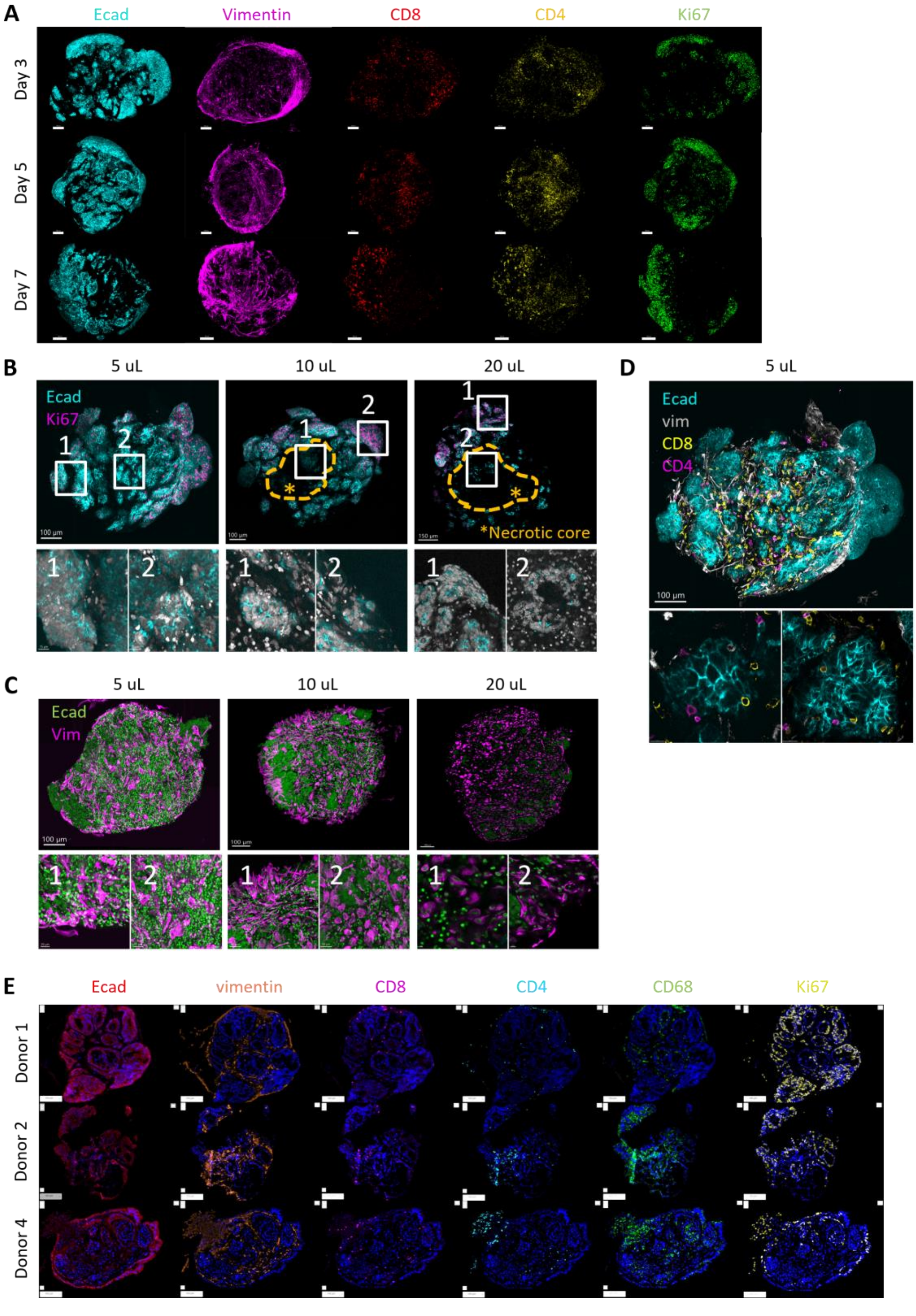

Fig. S1: Characterization of assembloid growth and inter-patient heterogeneity.
(A) Immunofluorescence staining of assembloids over time (days 3, 5, and 7) shows the spatial distribution of the different cell types. Scale bars: 100 µm. (B) Cell number experiments investigating assembloid volumes (5 µL, 10 µL, and 20 µL) revealed that larger assembloids (10 µL and 20 µL) developed necrotic cores (dashed yellow outlines). Scale bars: 100 or 150 µm. (C) Morphological changes in cancer-associated fibroblasts (CAFs) were observed in larger assembloids (10 µL and 20 µL) compared to smaller ones (5 µL). Insets (1 and 2) highlight differences in CAF morphology and epithelial organization. Scale bars: 100 or 150 µm. (D) Immune cell localization was analyzed in 5 µL assembloids by immunofluorescence. Most immune cells were found in CAF-rich regions, although some infiltrated epithelial regions. Scale bar: 100 µm. (E) Immunofluorescence staining of assembloids derived from different patients shows variations in cellular morphology and organization. Scale bars: 100 or 200 µm.

SUPPLEMENTARY FIGURE 2

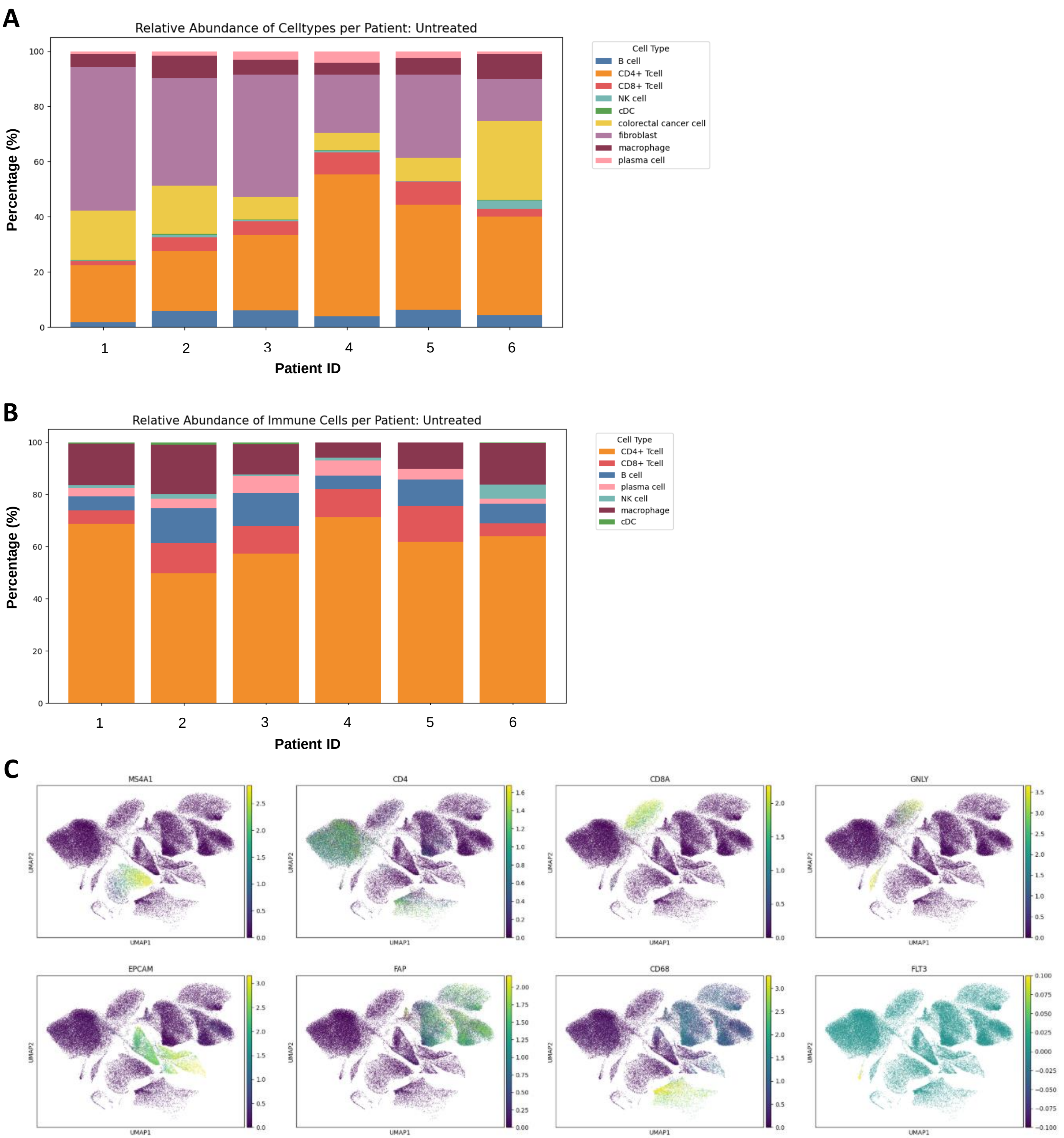

Fig. S2: Cellular composition and marker expression in assembloids across patients.
(A) Stacked bar plot showing the relative abundance of cell types in untreated assembloids derived from different patients. Variability in cellular composition is observed across patients. n = 6 independent patients (biological replicates). (B) Stacked bar plot showing the relative abundance of immune cells in untreated assembloids derived from different patients. n = 6 independent patients (biological replicates). (C) UMAP plots showing the expression of key marker genes across cell populations in untreated assembloids. Marker genes include MS4A1 (B cells), CD4 (CD4+ T cells), CD8A (cytotoxic CD8+ T cells), GNLY (natural killer cells), EPCAM (epithelial cells), FAP (CAFs), CD68 (macrophages), and FLT3 (dendritic cells).

FIGURE 2

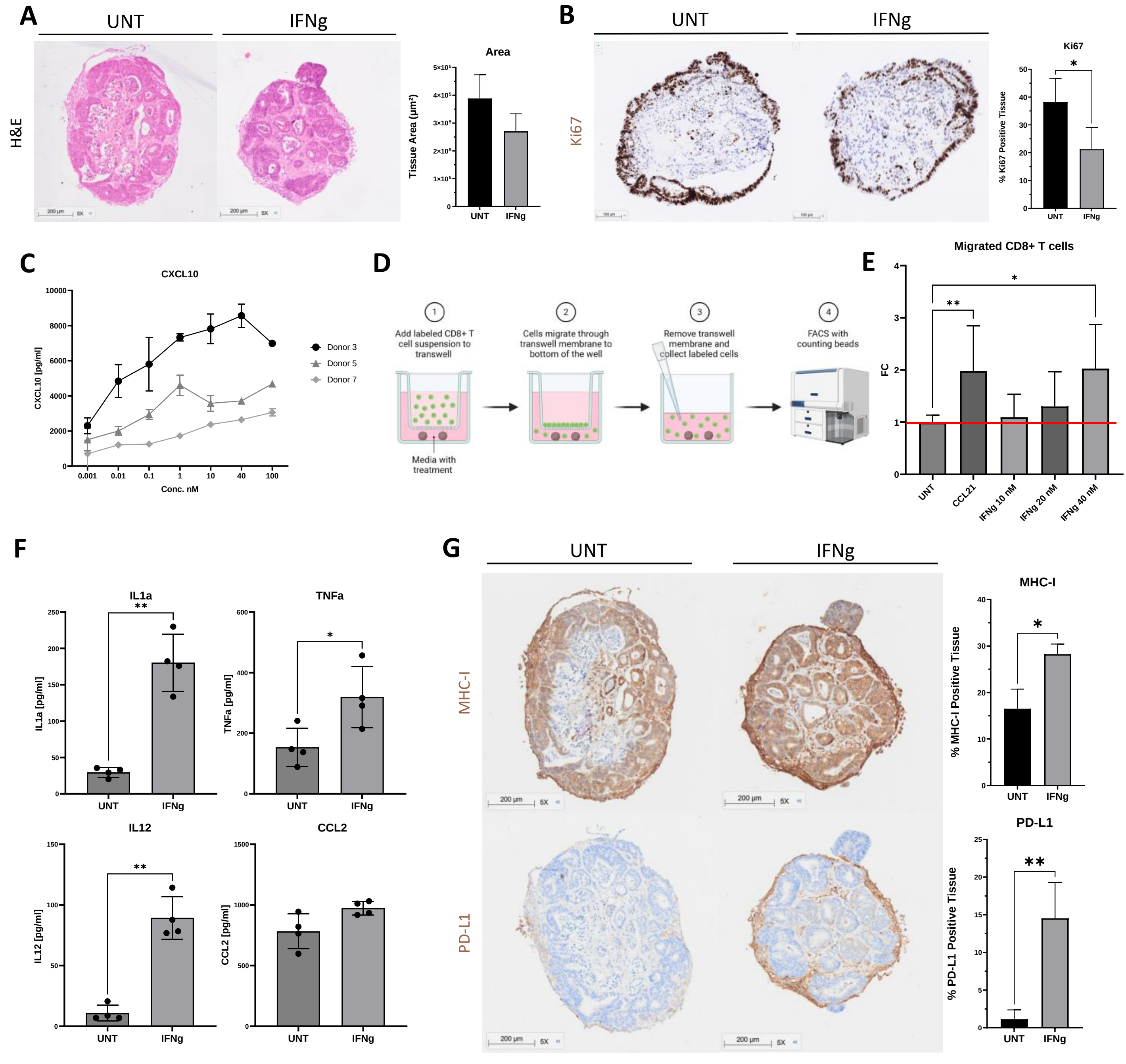

Fig. 2: Evaluation of the 3D immunocompetent model to predict IFNg response.
(A) Hematoxylin and eosin staining of untreated (UNT) and IFNg-treated assembloid shows a reduction in total tissue area following IFNg treatment (40 nM). Quantification of total area is shown as a bar graph. Data are presented as mean ± SD (n = 3 independent patients (biological replicates)). Scale bars, 200 µm. (B) Representative immunohistochemistry staining for Ki67 reveals a reduction in proliferative activity in IFNg-treated (40 nM) assembloids compared to untreated controls. Quantification of Ki67-positive cells is shown as a bar graph with mean ± SD (n = 4 independent patients (biological replicates), paired t-test). Scale bars, 100 µm. (C) Quantification of CXCL10 secretion in response to increasing doses of IFNg, as measured by ELISA in conditioned media. Each line represents an independent patient-derived assembloid line (Donor 3, 5 and 7). Data are shown as mean ± SD. (D) Schematic representation of a transwell migration assay to assess CD8+ T-cell recruitment. (E) Quantification of migrated CD8+ T cells in response to media with different treatments. IFNg-treated assembloids significantly enhanced CD8+ T-cell migration compared to untreated controls in a dose-dependent manner. CCL21 (0.1 ug/mL) was used as a positive control. Data are pooled from three independent patient-derived assembloid lines, normalized to baseline migration (red line) and displayed as mean ± SD, Mixed-effects model fitted by Restricted Maximum Likelihood (REML) with Dunnett's post-hoc test. (F) Multiplex cytokine analysis reveals increased levels of inflammatory and immune-modulating cytokines, including IL-1α, TNFα, CCL2, and IL-12, in conditioned media from IFNg-treated (40 nM) assembloids. Data are presented as mean ± SD (n = 4 independent patients (biological replicates), paired t-test). (G) Immunohistochemical staining for MHC-I and PD-L1 demonstrates upregulation of these immune markers in IFNg-treated (40 nM) assembloids. Corresponding quantification of MHC-I and PD-L1 expression is represented as the percentage of positive-stained area (mean ± SD, n = 3 independent patients (biological replicates), paired t-test). Scale bars, 200 µm. Figure (D) created with BioRender.com.

SUPPLEMENTARY FIGURE 3

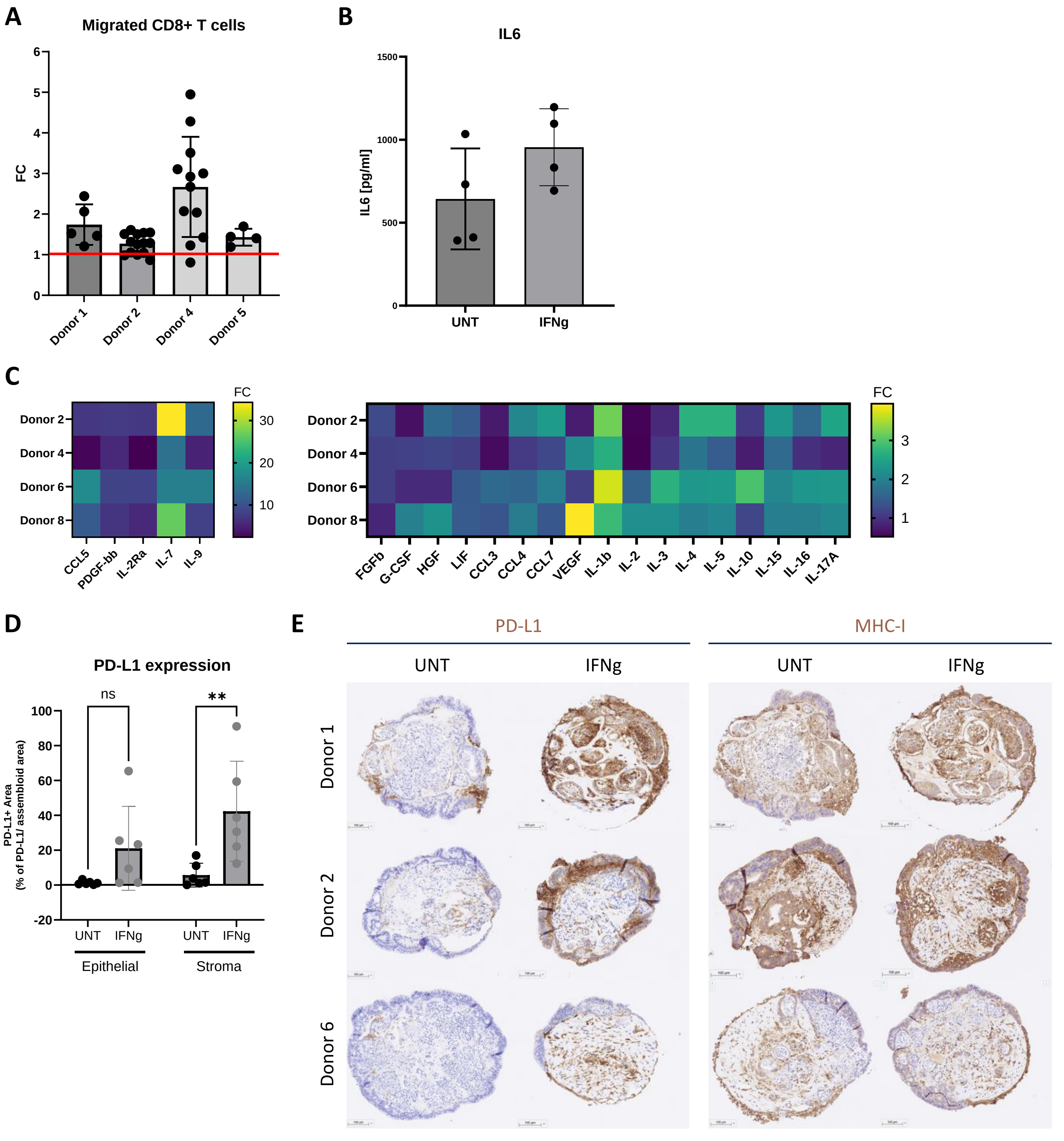

Fig. S3: Patient-specific responses to IFNg treatment in the assembloids.
(A) Transwell migration assay showing CD8+ T-cell recruitment across assembloids derived from different patients. IFNg treatment (40 nM) enhanced CD8+ T-cell migration, with variability in response observed between patients. Data are presented as mean ± SD. (B) Cytokine analysis of IL-6 levels in conditioned media from untreated and IFNg-treated (40 nM) assembloids. Data are presented as mean ± SD (n = 4 independent patients (biological replicates)). (C) Heatmap showing the levels of additional cytokines and growth factors in conditioned media from assembloids derived from different patients. IFNg treatment (40 nM) induced cytokine production, with distinct patterns across patients. (D) Quantification of PD-L1 expression in assembloids represented as the percentage of positive-stained area (mean ± SD, n = 6 independent patients (biological replicates), two-way ANOVA with Fisher's LSD test). (E) Immunohistochemical staining for PD-L1 and MHC-I in assembloids from different patients. IFNg treatment (40 nM) upregulated PD-L1 and MHC-I expression in a patient-specific manner. Scale bars: 100 µm.

FIGURE 3

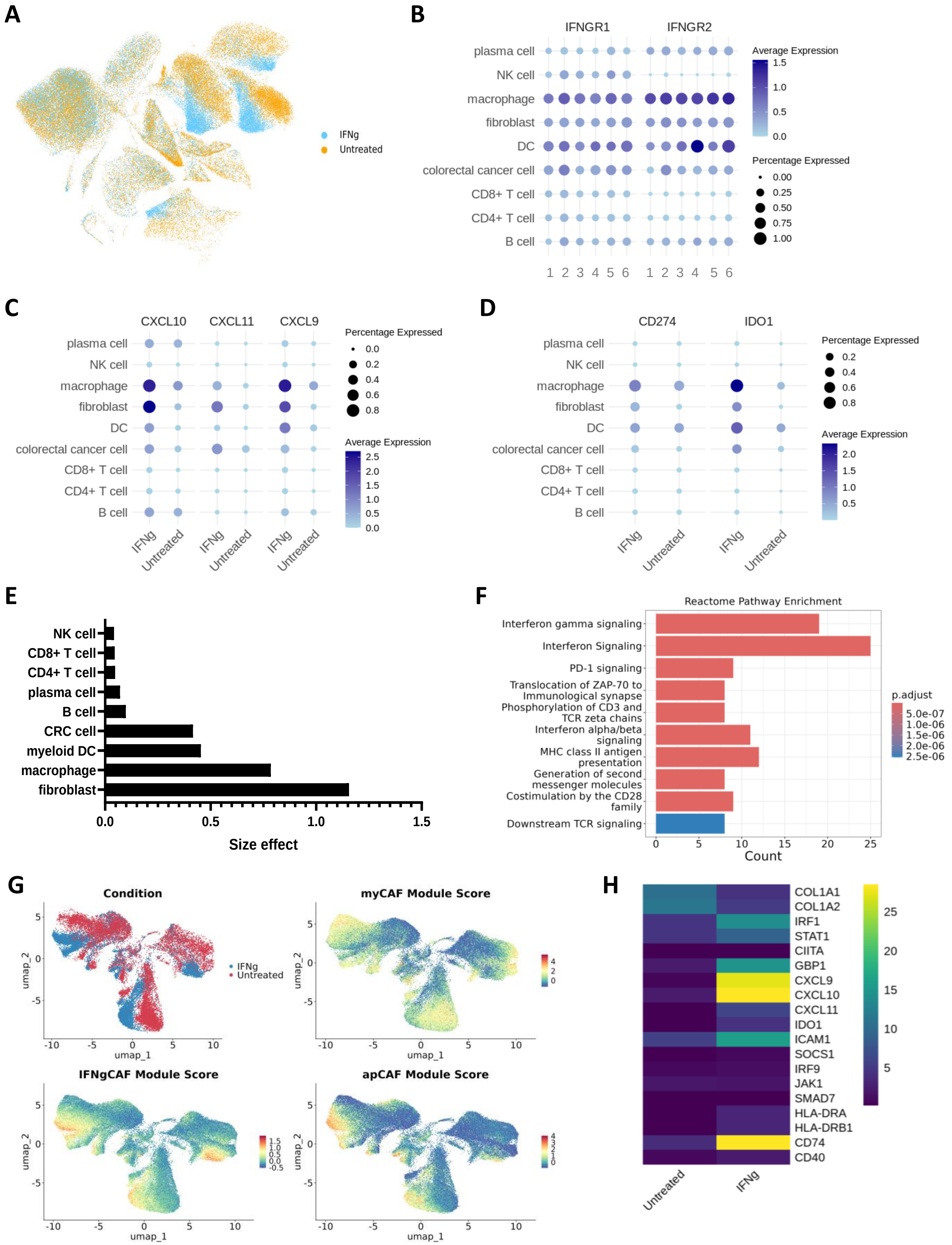

A
IFNg
Untreated
B
IFNGR1
IFNGR2
plasma cell
NK cell
macrophage
fibroblast
DC
colorectal cancer cell
CD8+ T cell
CD4+ T cell
B cell
1 2 3 4 5 6
Average Expression
Percentage Expressed
C
CXCL10
CXCL11
CXCL9
D
CD274
IDO1
E
NK cell
CD8+ T cell
CD4+ T cell
plasma cell
B cell
CRC cell
myeloid DC
macrophage
fibroblast
Size effect
F
Reactome Pathway Enrichment
Interferon gamma signaling
Interferon Signaling
PD-1 signaling
Translocation of ZAP-70 to Immunological synapse
Phosphorylation of CD3 and TCR zeta chains
Interferon alpha/beta signaling
MHC class II antigen presentation
Generation of second messenger molecules
Costimulation by the CD28 family
Downstream TCR signaling
Count
p.adjust
G
Condition
myCAF Module Score
IFNgCAF Module Score
apCAF Module Score
umap_1
umap_2
H
COL1A1
COL1A2
IRF1
STAT1
CIITA
GBP1
CXCL9
CXCL10
CXCL11
IDO1
ICAM1
SOCS1
IRF9
JAK1
SMAD7
HLA-DRA
HLA-DRB1
CD74
CD40
Untreated
IFNg

Fig. 3: Cell-type-specific responses to IFNg treatment reveal CAF phenotypic transition to apCAFs in assembloids. (A) UMAP showing cell clusters under untreated and IFNg-treated (40 nM) conditions, with color-coded treatment groups. Treatment with IFNg results in distinct transcriptional changes in specific cell populations. (B) Dot plot displaying the expression of IFNGR1 and IFNGR2 across cell types, highlighting the presence of IFNg receptors in various populations. (C) Dot plot showing the expression of chemokines in response to IFNg treatment across cell types. (D) Dot plot illustrating the expression of immune suppressive factors in different cell types following IFNg treatment. (E) Bar plot quantifying the size effect of IFNg treatment on gene expression changes across cell types, with fibroblasts showing the largest response. (F) Reactome pathway enrichment analysis in fibroblasts reveals activation of pathways associated with interferon signaling, antigen presentation, and immune response regulation. (G) UMAP feature plots showing module scores myCAF, ifngCAF, and apCAF signatures across fibroblasts, demonstrating functional changes in fibroblast subsets. (H) Heatmap illustrating the expression of key interferon response genes in untreated and IFNg-treated assembloids.

SUPPLEMENTARY FIGURE 4

A

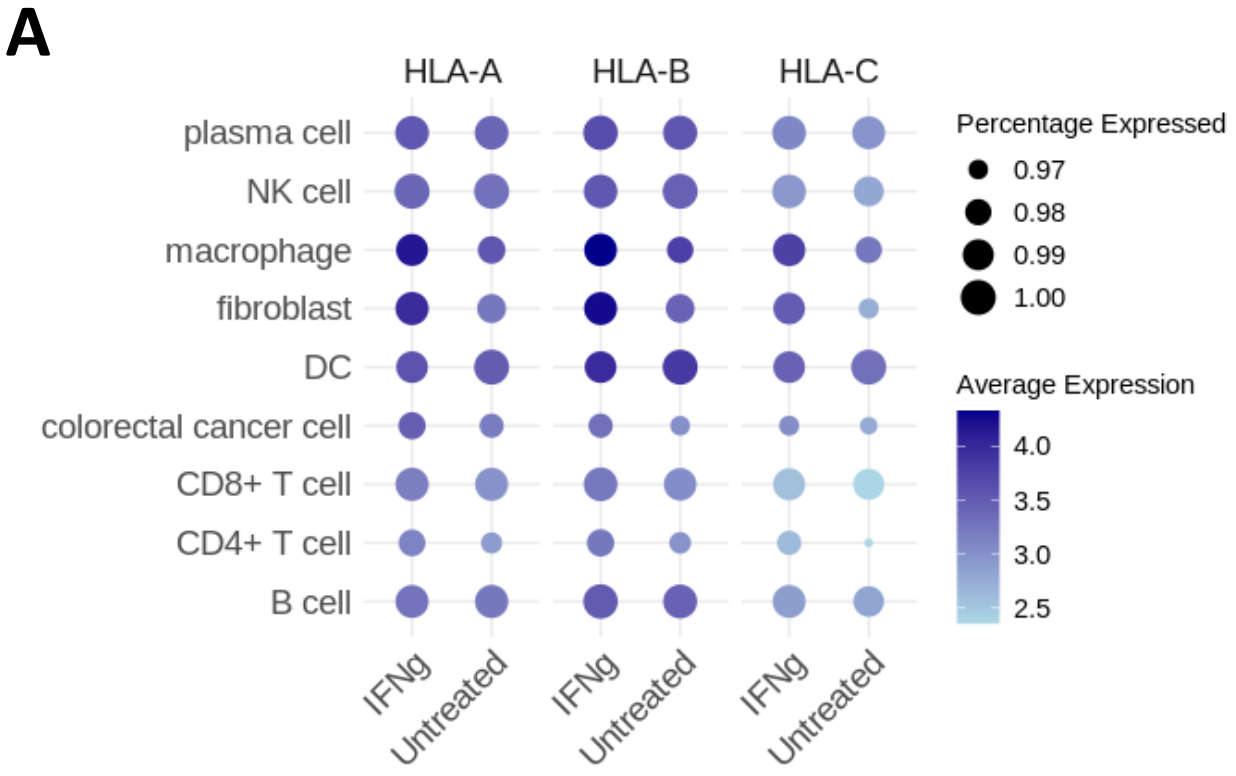

Fig. S4: Dot plot showing the expression of MHC-I genes in response to IFNg treatment across cell types.

SUPPLEMENTARY FIGURE 5

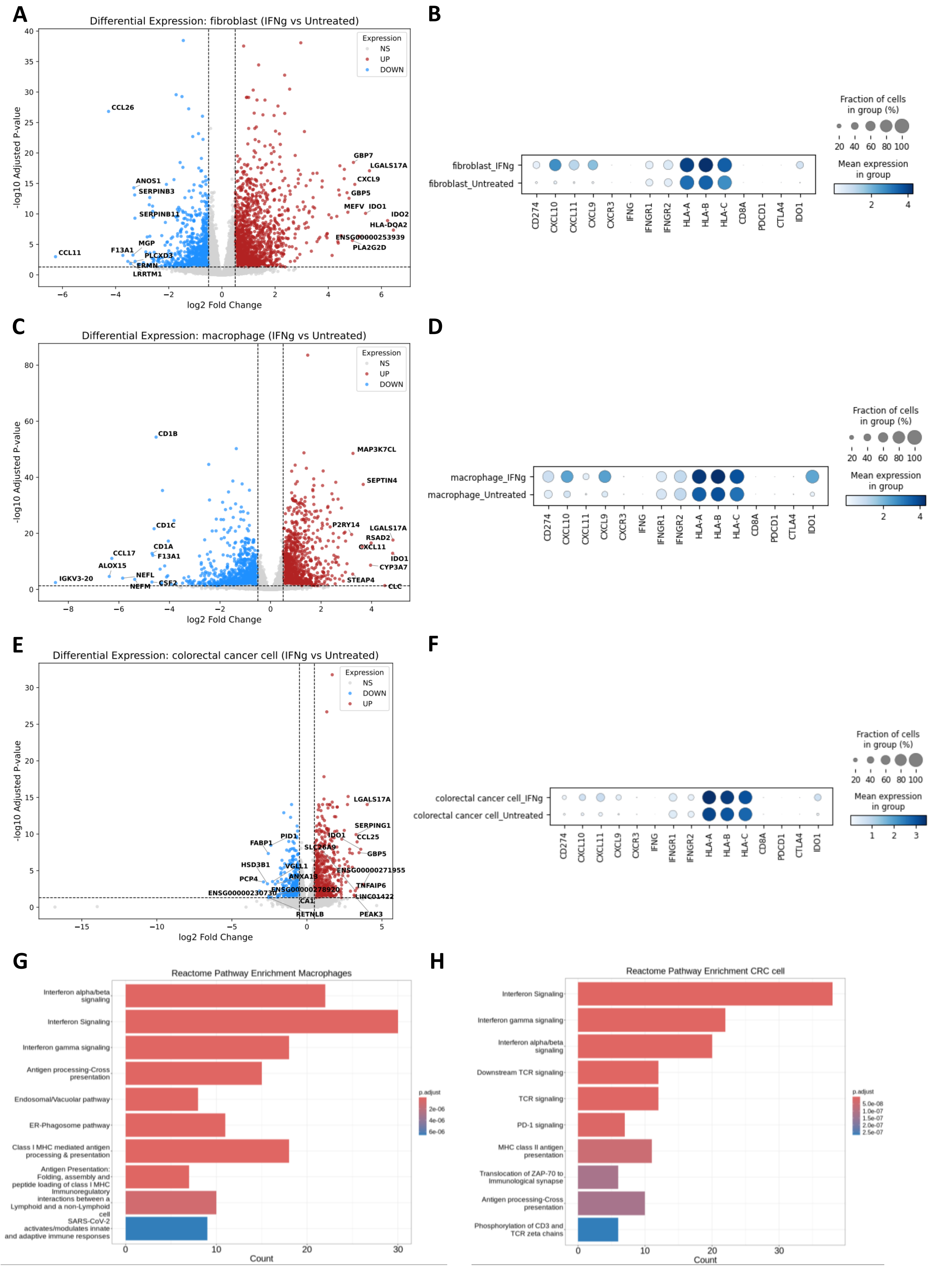

Fig. S5: Cell-type-specific differential expression and pathway enrichment profiling following IFNg exposure in assembloids.
(A) Volcano plot displaying differential gene expression between IFNg-treated and untreated conditions across fibroblasts. Significantly upregulated genes are indicated in red, significantly downregulated genes in blue, and non-significant changes in gray. (B) Dot plot illustrating the expression levels and distribution of selected IFNg-responsive genes in fibroblasts, comparing untreated and IFNg-treated conditions. Node size indicates the fraction of cells expressing the target gene, while color intensity represents the mean expression level within the group. (C) Volcano plot displaying differential gene expression between IFNg-treated and untreated conditions across macrophages. (D) Dot plot illustrating the expression levels and distribution of selected IFNg-responsive genes in macrophages, comparing untreated and IFNg-treated conditions. (E) Volcano plot displaying differential gene expression between IFNg-treated and untreated conditions across colorectal cancer cells. (F) Dot plot illustrating the expression levels and distribution of selected IFNg-responsive genes in colorectal cancer cells, comparing untreated and IFNg-treated conditions. (G, H) Reactome pathway enrichment analysis of the significantly altered genes following IFNg treatment in macrophages (G) and colorectal cancer cells (H). Highly enriched pathways in both compartments include "Interferon Signaling" and "Interferon gamma signaling". Bar length represents the count of genes associated with each biological pathway, and the color gradient indicates the adjusted p-value.

SUPPLEMENTARY FIGURE 6

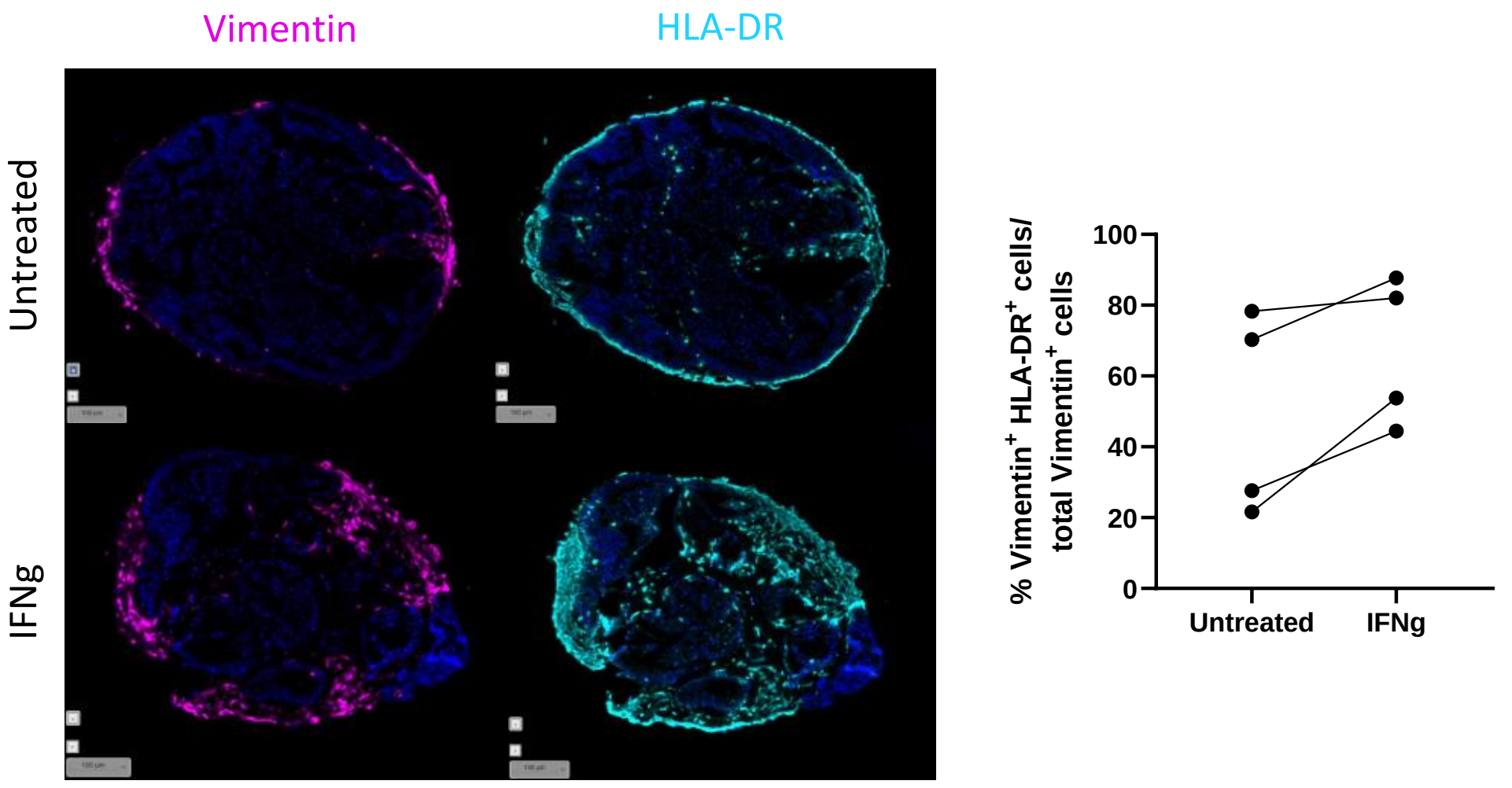

Fig. S6: Protein-level validation of the IFNg-induced apCAF phenotype in assembloids.
(Left) Representative multiplex immunofluorescence images of FFPE assembloid sections under untreated (top) and IFNg-treated (bottom) conditions, showing Vimentin (CAF marker, magenta) and HLA-DR (MHC-II, cyan). (Right) Quantification of the percentage of $Vimentin^{+}$ $HLA\text{-}DR^{+}$ double-positive cells out of total $Vimentin^{+}$ cells in untreated versus IFNg-treated conditions. Data points represent individual patient lines (n = 4 independent patients (biological replicates), paired analysis). Scale bars, 100 µm.

FIGURE 4

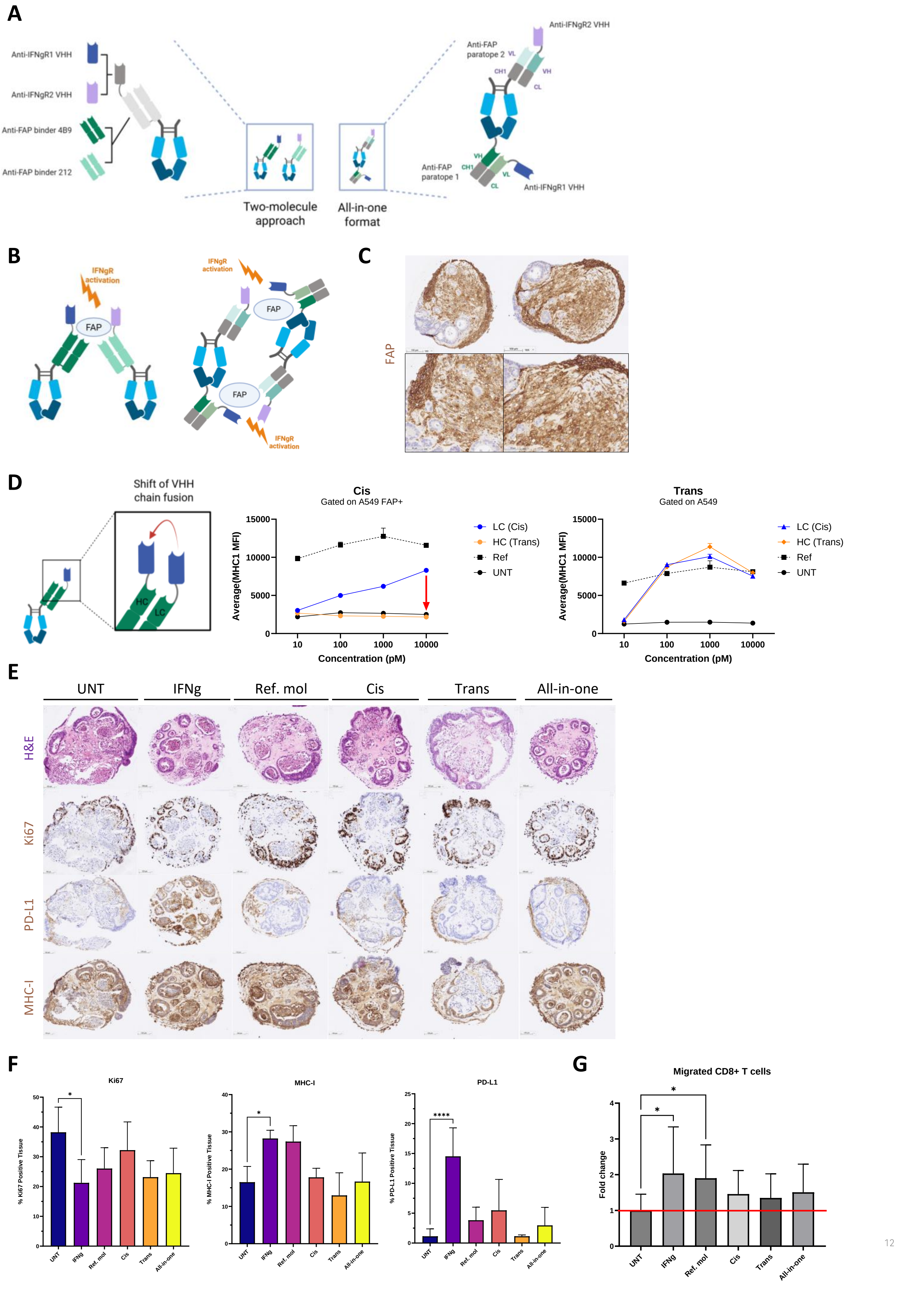

A
Anti-IFNgR1 VHH
Anti-IFNgR2 VHH
Anti-FAP binder 4B9
Anti-FAP binder 212
Two-molecule approach
All-in-one format
Anti-FAP paratope 2
Anti-FAP paratope 1
Anti-IFNgR1 VHH
B
IFNgR activation
FAP
C
FAP
D
Shift of VHH chain fusion
HC
LC
Cis
Gated on A549 FAP+
Trans
Gated on A549
Average(MHC1 MFI)
Concentration (pM)
LC (Cis)
HC (Trans)
Ref
UNT
E
UNT
IFNg
Ref. mol
Cis
Trans
All-in-one
H&E
Ki67
PD-L1
MHC-I
F
Ki67
% Ki67 Positive Tissue
MHC-I
% MHC-I Positive Tissue
PD-L1
% PD-L1 Positive Tissue
G
Migrated CD8+ T cells
Fold change

Fig. 4: Development and evaluation of novel FAP-targeting molecules in assembloids.
(A) Schematic of two FAP-targeting strategies: the two-molecule approach (separate FAP binder and anti-IFNgR1 or anti-IFNgR2 VHH) and the all-in-one format (containing both IFNgR VHHs). (B) Illustration of the mode of action of the FAP-targeting construct binding to FAP-expressing cells and subsequent IFNgR activation. (C) Immunohistochemistry showing FAP expression in the assembloids. Scale bars, 100 µm. (D) Schematic of VHH chain fusion shift and quantification of binding affinity in cis and trans configurations (Left). Graphs show dose-dependent MFI (mean fluorescence intensity) values for MHC-I for each treatment (cis and trans combination, reference molecule and untreated control) (Right). (E) Representative H&E and immunohistochemical staining of assembloids treated with IFNg, reference molecule, cis, trans, and all-in-one FAP-IFNg molecules, all in 100 nM. Scale bars, 100 µm. (F) Quantification of Ki67, MHC-I, and PD-L1 staining in assembloids, highlighting differences in immune marker expression and proliferative activity upon treatment. Data are presented as mean ± SD (n = 3 or 4 independent patients (biological replicates), one-way ANOVA with Dunnett's multiple comparison test). (G) Transwell migration assay showing enhanced CD8+ T-cell recruitment in response to FAP-IFNg-treated assembloids. Data represented as fold change, normalized to baseline migration (red line). Fold change of the ratio of cell count, normalized to bead counts. Data are presented as mean ± SD (n = 3 independent patients (biological replicates), one-way ANOVA with Tukey's multiple comparison test). Figure A, B and left panel of D created with BioRender.com.

A

ID	FAP binder	Chain fusion	VHH
Reference	/	/	R1+R2
P1AI0831	4B9	LC	R1
P1AI0066	212	HC	R2
P1AI0160	4B9	HC	R1
All-in -one	4B9 + 212	LC	R1+R2

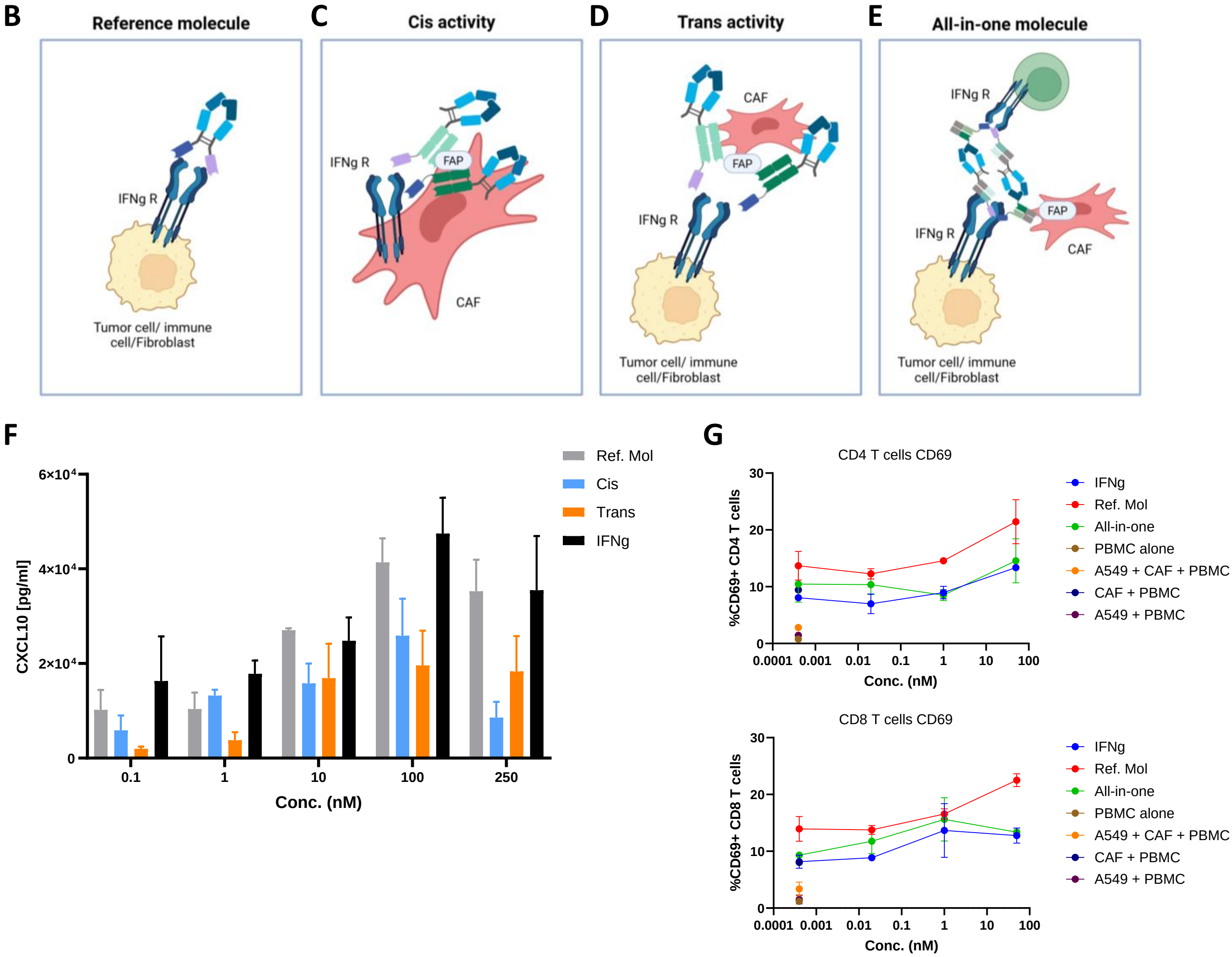

Fig. S7: Schematic representation of the FAP-IFNg molecules and their modes of activity.
(A) Table summarizing FAP binders, chain fusions (HC/LC), and VHH constructs of the FAP-IFNg molecules, including the reference molecule. (B–E) Schematic representations of the engineered FAP-IFNg molecules and their distinct spatial mechanisms of receptor activation. (B) A standard non-targeted reference molecule designed to indiscriminately engage the IFNgR on responsive cells (e.g., tumor cells, immune cells, or fibroblasts). (C) The two-molecule approach demonstrating cis activity, where the constructs bind to FAP and activate the IFNgR on the surface of the same cancer-associated fibroblast (CAF). (D) The two-molecule approach demonstrating trans activity, where the constructs anchor to FAP on a CAF and bridge across to activate the IFNgR on a neighboring cell. (E) An "all-in-one" molecule that integrates anti-FAP binders and anti-IFNgR VHHs into a single unified format to engage both IFNgR targets simultaneously. (F) Dose-response evaluation of CXCL10 secretion (pg/mL) in assembloids treated with increasing concentrations (0.1 to 250 nM) of recombinant IFNg, the non-targeted reference molecule (Ref. Mol), the targeted Cis combination, and the Trans combination. Data are presented as mean ± SEM (n = 3 independent patients (biological replicates)). (G) Flow cytometric quantification of CD69 surface expression on $CD4^+$ T cells (top) and $CD8^+$ T cells (bottom) following stimulation across a concentration titration (0.001–50 nM) of recombinant IFNg, reference molecule (Ref. Mol), and targeted construct (All-in-one). Baseline activation for unstimulated control co-culture conditions are plotted at baseline: PBMC alone, CAF + PBMC, A549 + PBMC, and A549 + CAF + PBMC co-cultures. Data are presented as mean ± SD (n = 3 technical replicates).
